# Time-calibrated Milankovitch cycles for the late Permian

**DOI:** 10.1038/ncomms3452

**Published:** 2013-09-13

**Authors:** Huaichun Wu, Shihong Zhang, Linda A. Hinnov, Ganqing Jiang, Qinglai Feng, Haiyan Li, Tianshui Yang

**Affiliations:** 1 State Key Laboratory of Biogeology and Environmental Geology, China University of Geosciences, Beijing 100083, China; 2 School of Ocean Sciences, China University of Geosciences, Beijing 100083, China; 3 Department of Earth and Planetary Sciences, Johns Hopkins University, Baltimore, Maryland 21218, USA; 4 Department of Geoscience, University of Nevada, Las Vegas, Nevada 89154, USA; 5 State Key Laboratory of Geological Processes and Mineral Resources, China University of Geosciences, Wuhan 430074, China

## Abstract

An important innovation in the geosciences is the astronomical time scale. The astronomical time scale is based on the Milankovitch-forced stratigraphy that has been calibrated to astronomical models of paleoclimate forcing; it is defined for much of Cenozoic–Mesozoic. For the Palaeozoic era, however, astronomical forcing has not been widely explored because of lack of high-precision geochronology or astronomical modelling. Here we report Milankovitch cycles from late Permian (Lopingian) strata at Meishan and Shangsi, South China, time calibrated by recent high-precision U–Pb dating. The evidence extends empirical knowledge of Earth’s astronomical parameters before 250 million years ago. Observed obliquity and precession terms support a 22-h length-of-day. The reconstructed astronomical time scale indicates a 7.793-million year duration for the Lopingian epoch, when strong 405-kyr cycles constrain astronomical modelling. This is the first significant advance in defining the Palaeozoic astronomical time scale, anchored to absolute time, bridging the Palaeozoic–Mesozoic transition.

## Abstract (web summary)

The astronomical time scale is an essential geochronological tool, but is presently limited to the Cenozoic and Mesozoic eras. Here, Wu *et al.* time-calibrate Milankovitch cycles identified in strata from South China and extend this essential tool into the late Permian.

## Graphical abstract

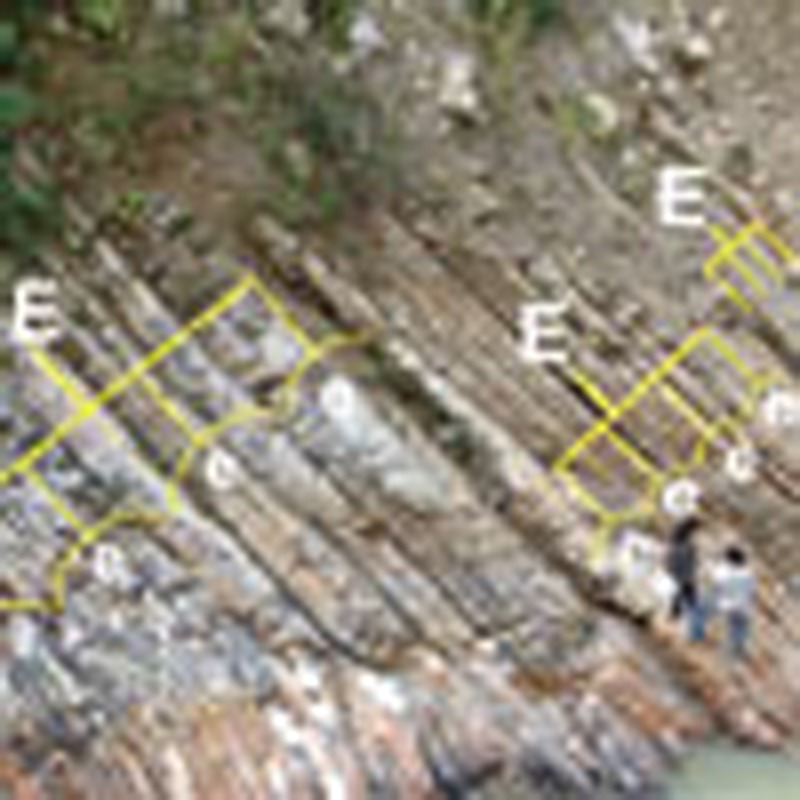

The cyclostratigraphic record of astronomically forced climate change^1^, when tuned to an astronomical solution, provides a high-resolution astronomical time scale (ATS)^2^,^3^,^4^. The construction of the ATS is well underway for the Cenozoic–Mesozoic eras (0–252 million years ago (Ma))^5^,^6^, and is increasingly being used to inter-calibrate geochronology^7^,^8^,^9^. Prospects for a Palaeozoic ATS are excellent^6^, but among the challenges is the lack of an accurate astronomical solution or confirmation of astronomically forced sedimentary cycles constrained by high-precision geochronology. Palaeozoic (and earlier) time remains in the purview of empirically determined astronomical forcing^10^,^11^.

Recently, high-precision U–Pb ages^12^ were obtained from Upper Permian sedimentary sections at Meishan and Shangsi, South China^13^,^14^. These sections, separated by ~1,350 km, represent late Permian depositional systems in the eastern Paleo–Tethys Ocean (Supplementary Fig. S1). At Meishan, the Changxing Formation was deposited in carbonate platform/slope environments^13^,^14^. The Permian–Triassic boundary (PTB) is at the base of bed 27c^13^. At Shangsi, the carbonate-rich Wujiaping Formation was deposited in a deepening platform, and the overlying Dalong Formation in slope/basinal environments, with carbonate increasingly replaced by clay deposition^15^. The section is correlated to Meishan with biostratigraphy and U–Pb dating^12^; the PTB is placed at the bed 28b/28c boundary^12^. The carbon isotope (*δ*^13^C_carb_) record at Shangsi is similar to that at Meishan and other sections^12^,^13^,^16^,^17^. The end-Permian mass extinctions are recorded in both sections^12^,^18^,^19^.

Here together with the new U–Pb dates^12^,^18^, we study the stratigraphic cyclicity in the Shangsi and Meishan sections and present evidence for Milankovitch cycles in the late Permian, leading up to and through the greatest mass extinctions^19^,^20^,^21^,^22^,^23^, and linked to the 250-Ma terminus of astronomical solutions^10^,^11^. We identify periods of obliquity and precession terms that are consistent with a 22-h length-of-day predicted for 250 Ma. According to the ATS at the Shangsi section, the duration of the Lopingian epoch is 7.793 Myr, and the mass extinction interval is 380 kyr.

## Results

### Rock magnetic stratigraphic series

**Figure 1:**
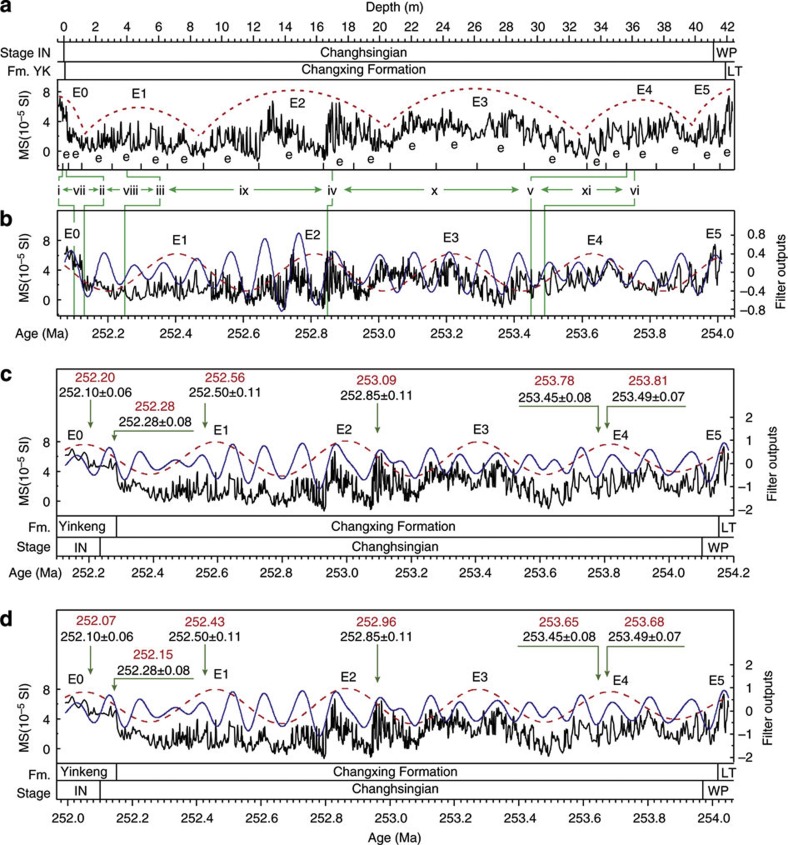
Cyclostratigraphy of the Meishan section. (**a**) MS series of the Meishan section. The interpretation of 405-kyr-long eccentricity (E) and ~100-kyr-short eccentricity (e) cycles is based on the spectral analysis (Figure 3a). (**b**) U–Pb ages (green lines) calibrated MS time series with 405-kyr (red) and 100-kyr (blue) Gauss filter outputs, with passbands of 0.002469±0.00025 and 0.01±0.002 cycles per kyr respectively. The U–Pb ages are from ref. 12. The roman numerals i, ii, iii, iv, v and vi represent the ages of 252.10±0.06, 252.28±0.08, 252.50±0.11, 252.85±0.11, 253.45±0.08 and 253.49±0.07 Ma at different depths. The numbers vii, viii, ix, x and xi represent the U–Pb age-calibrated durations of 0.18±0.1, 0.22±0.14, 0.35±0.16, 0.60±0.14 and 0.04±0.11 Myr, respectively. Uncertainties are calculated by error propagation. (**c**) 405-kyr-tuned MS time series with 405-kyr (red) and 100-kyr (blue) Gauss filter outputs with passbands of 0.002469±0.00025 and 0.01±0.0035 cycles per kyr, respectively. The paired red and black numbers are 405-kyr-tuned and U–Pb ages for comparison, labelled in ‘Ma’. (**d**) Adjusted 405-kyr-tuned MS time series based on the synchrony of end-Permian mass extinction in South China and the La2010d solution from Shangsi section (see main text for explanation). The paired red and black numbers are adjusted 405-kyr-tuned ages and corresponding U–Pb ages in Ma. Fm., formation; IN, Induan; LT, Longtan Formation; WP, Wuchiapingian; YK, Yinkeng Formation.

**Figure 2:**
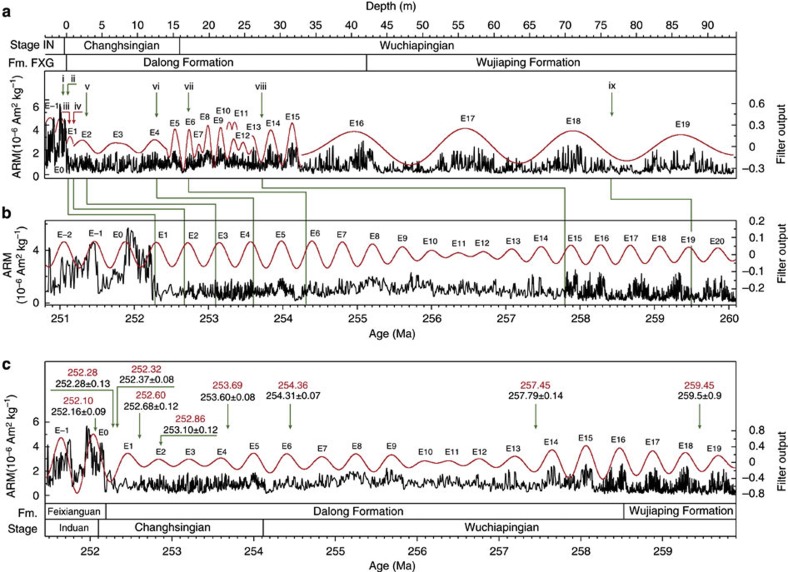
Cyclostratigraphy of the Shangsi section. (**a**) ARM series of the Shangsi section. The interpreted 405-kyr cycles (red) were extracted using Gauss filters with passbands of 0.22±0.08 cycles per m (1–14 m), 0.6±0.3 cycles per m (14–26 m), 0.4±0.12 cycles per m (26–33 m) and 0.06±0.02 cycles per m (33–93.6 m). The roman numbers i, ii, iii, iv, v, vi, vii, viii and ix represent the U–Pb ages of 252.16±0.09, 252.28±0.13, 252.37±0.08, 252.68±0.12, 253.10±0.12, 253.60±0.08, 254.31±0.07, 257.79±0.14 and 259.5±0.9 Ma at different depths, respectively. The former eight U–Pb ages are from ref. 12 and the last (259.5±0.9 Ma) is from ref. 18. (**b**) U–Pb ages (green lines) calibrated ARM time series with 405-kyr signal (red) extracted using a Gauss filter with a passband of 0.002469±0.00015 cycles per kyr. (**c**) 405-kyr-tuned ARM time series with 405-kyr filter output (red) extracted using a Gauss filter with a passband of 0.002469±0.00075 cycles per kyr. The paired red and black numbers are 405-kyr-tuned ages and U–Pb ages for comparison, labelled in Ma. Fm., formation; FXG=Feixianguan Formation; IN, Induan.

We collected high-resolution series of magnetic susceptibility (MS) at Meishan and anhysteretic remanent magnetization (ARM) at Shangsi, showing significant cyclic variations (Figs 1 and 2; Supplementary Figs S2–S7). High MS and ARM values occur in lithologies with high clay or mud content, and low values occur in carbonate-rich strata. The abrupt increase of MS and ARM in Lower Triassic strata is from increased detrital input from elevated continental weathering following the mass extinctions^24^.

### Cycle analysis and time calibration

**Figure 3:**
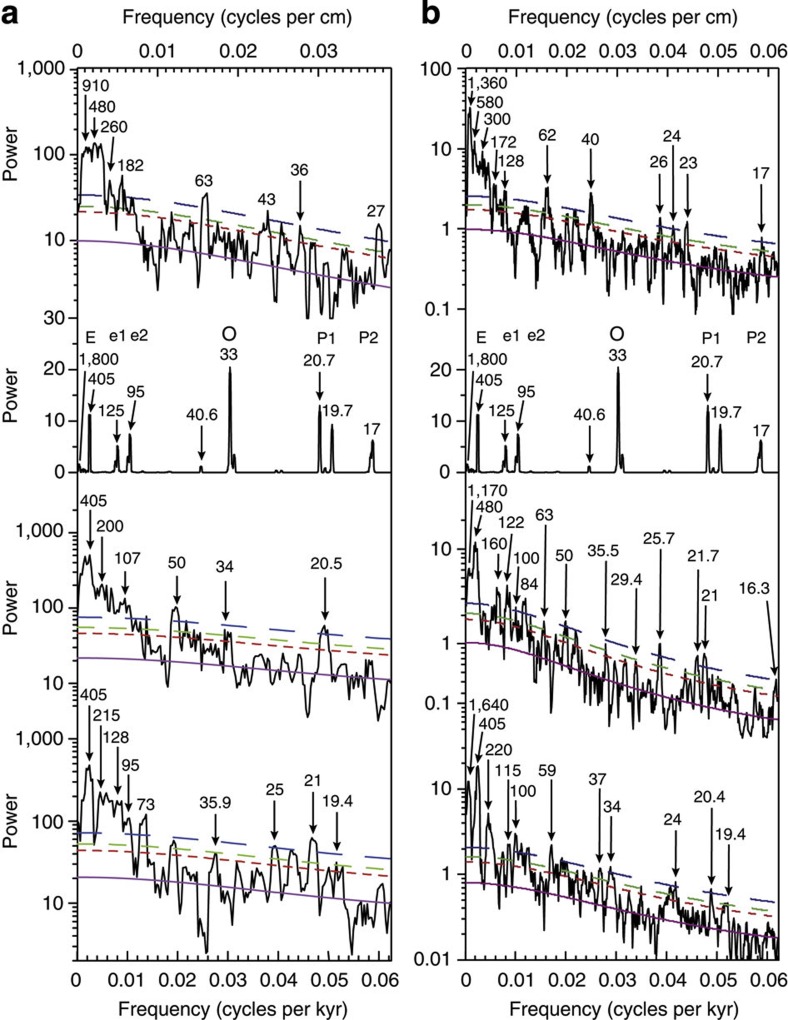
Spectral analysis. (**a**) 2*π* power spectra of the Meishan MS series, from the top: stratigraphic series, La2004 ETP time series from 240–249 Ma, U–Pb age-calibrated time, 405-kyr-tuned time. (**b**) 3*π* power spectra of the Shangsi ARM series, from the top: stratigraphic domain, La2004 ETP time series from 240–249 Ma, U–Pb age-calibrated time, 405-kyr-tuned time. The blue, green and red curves indicate 99, 95 and 90% confidence limits. The purple curve ‘M’ indicates the smoothed, fitted red-noise spectrum. The letters E, e, O and P represent the 405-kyr eccentricity, short (~100-kyr) eccentricity, obliquity and precession orbital parameters. Significant peaks are labelled in centimeters for the stratigraphic spectra (which refer to the upper *x* axis) and in kilo years for the others (which refer to the lower *x* axis).

The Meishan MS stratigraphic spectrum has numerous peaks suggesting a variable sediment accumulation rate (Fig. 3a, Supplementary Fig. S8). The U–Pb age-constrained^12^ MS spectrum has peaks at periods of 405-, 107- and 20.5-kyr above 99% confidence and a 34-kyr peak above 95% confidence (Fig. 3a, Supplementary Fig. S9). The Shangsi ARM stratigraphic spectrum also shows numerous peaks signalling a variable sediment accumulation rate (Fig. 3b, Supplementary Fig. S10) keyed with lithological changes^25^ (Supplementary Fig. S11). The U–Pb age-constrained^12^,^18^ ARM spectrum has peaks at periods of 1,170, 480, 122, 100, 84, 50, 35.5, 29.4, 21.7 and 21 kyr (Fig. 3b, Supplementary Fig. S12). The periodicities in both spectra are consistent with astronomical modelling^10^ and show focusing of power in the precession band.

Strong ~405 kyr cycles predominate in both U–Pb age-calibrated series, which we interpret as evidence of forcing from Earth’s 405-kyr orbital eccentricity cycle. This cycle originates from interaction between Venus and Jupiter orbital perihelia, and is stable over long timescales owing to the great mass of Jupiter. The 405-kyr cycle has been adopted as a ‘metronome’ for the astronomical tuning of the Cenozoic–Mesozoic stratigraphy^6^,^10^,^11^,^26^.

We applied the metronome concept to the MS and ARM series using the interpreted 405-kyr cycles and the U–Pb age of 252.28 Ma as an anchor point for both sections (Figs 1c and 2c; Supplementary Figs S6c and S7c; Supplementary Table S1). Most of the 405-kyr-tuned ages are consistent with the U–Pb ages. The 405-kyr-tuned MS spectrum has peaks at 405-, 128-, 95-, 35.9-, 25-, 21- and 19.4-kyr periods (Fig. 3a, Supplementary Fig. S13); the 405-kyr-tuned ARM spectrum has peaks at 1,640-, 405-, 220-, 115-, 100-, 59-, 34-, 24-, 20.4-, 19.4- and 13.9-kyr periods (Fig. 3b, Supplementary Fig. S14).

### Amplitude modulation analysis

**Figure 4:**
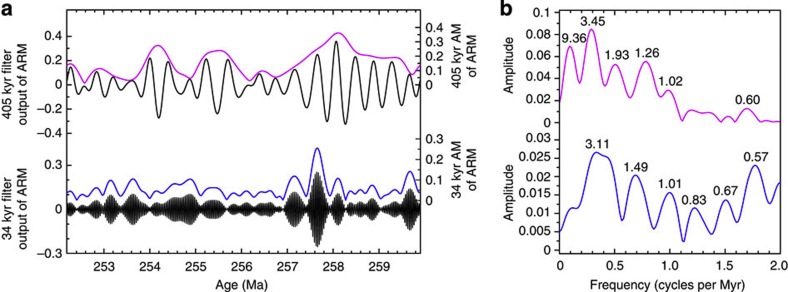
Long period AM analysis of Shangsi section ARM series. (**a**) 405-kyr eccentricity (black) and its AM (purple); 34-kyr obliquity (black) and its AM (blue). The 405- and 34-kyr cycles were extracted using Taner filter with passbands of 0.002469±0.0014 and 0.0291±0.0025 cycles per kyr, respectively. (**b**) 2*π* multitapered amplitude spectra of the AM series of interpreted 405-kyr eccentricity (upper, purple) and 34-kyr obliquity signals (lower, blue). Period peaks are labelled in Myr.

The amplitude modulations (AM) of the interpreted 405 and 34-kyr bands of the Shangsi ARM time series display long-period cycles (Fig. 4). The spectrum of the 405-kyr AM signal has significant peaks at 3.45, 1.93, 1.26 and 1.02 Myr. The spectrum of the 34-kyr AM signal has major peaks at 3.11 and 0.57 Myr.

## Discussion

Most of the cyclicity in the MS and ARM series is consistent with predicted early Triassic astronomical parameters^10^ (Fig. 3). In both sections, 34-kyr obliquity and ~21–19-kyr precession periods support the La2004 tidal dissipation model, which assumes an increasing length-of-day of 2.68 ms per century over 0–250 Ma, that is, a ~22-h-long late Permian day^10^.

**Figure 5:**
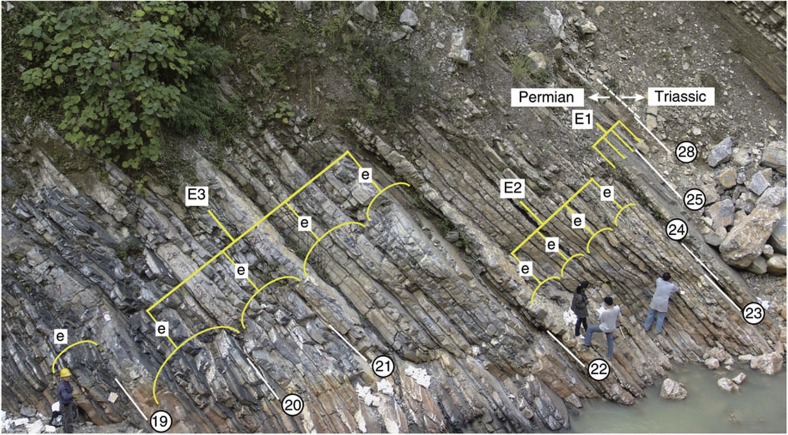
Photo of the Upper Changhsingian Dalong Formation at Shangsi section. Five thin precession-scale beds are bundled into 100-kyr eccentricity cycles (e) and four ~100-kyr cycles are bundled into 405-kyr eccentricity cycles (E). The ARM cycle interpretation is provided in Fig. 2, Supplementary Figs S4 and S5. Eccentricity maxima are recorded by pronounced, thin precession beds, whereas the eccentricity minima correlate to thick limestone beds. Circled numbers indicate bed numbers; white lines mark bed boundaries.

In the Upper Dalong Formation at Shangsi (Fig. 5, Supplementary Fig. S11), precession-scale carbonate-rich beds with prominent shaly intercalations formed during high eccentricity (high ARM), and thick limestones were deposited during low eccentricity (low ARM). This pattern is consistent with the theoretical precession index, in which cycles with the highest amplitudes (high eccentricity) have the shortest periods^27^. It suggests increased erosion and detrital sediment delivery (intensified hydrologic cycle) and higher ARM during high eccentricity, and less erosion and lower ARM during low eccentricity. We adopt the same interpretation for the Meishan section.

**Figure 6:**
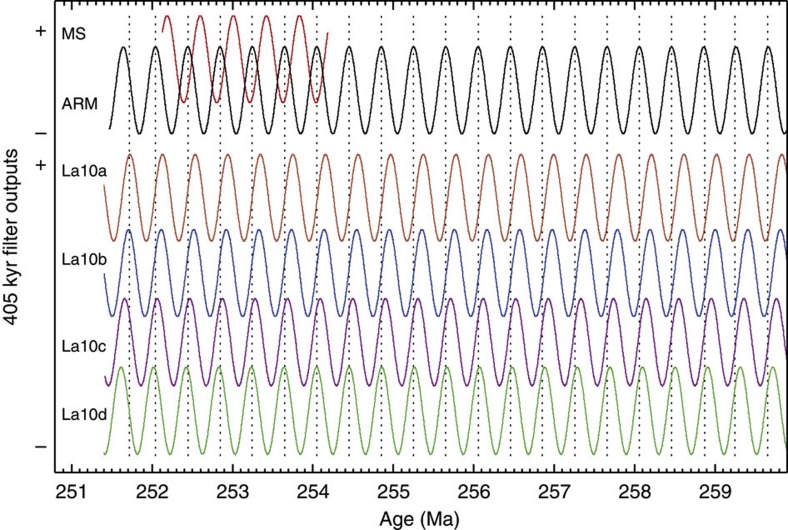
Comparison of late Permian 405-kyr cycles. The Meishan MS and Shangsi ARM series subjected to Taner filtering with a very narrow passband of 1/405.091±0.00001 cycles per kyr to isolate the 405-kyr cycles. The bottom four curves are 405-kyr eccentricity cycles extrapolated from 250 to 260 Ma, from La2010a–d eccentricity series (ref. 11) filtered by a very narrow passband of 1/405.091 kyr±0.000001 cycles per kyr. The dotted vertical lines mark the 405-kyr maxima of the Shangsi 405-kyr cycles to highlight the differences among the ARM, MS and La2010a–d 405-kyr eccentricity cycles.

The 405-kyr cycling allows extension of the 405-kyr metronome from 250 Ma into the Palaeozoic era. The 405-kyr cycles of the two sections (Fig. 6) show that Meishan cycles lag Shangsi cycles by ~150 kyr. The ARM and MS proxies are comparable (that is, Shangsi MS is phased with ARM, *cf.* ref. 17), and so, assuming that the 405-kyr cycles should be in phase between Meishan and Shangsi, there is an error in the anchor point linking the sections, that is, the 252.28-Ma U–Pb date.

To understand this discrepancy, we compare the Meishan and Shangsi 405-kyr cycles with La2010 (ref. 11) 405-kyr eccentricity cycles extrapolated from 250 Ma (the La2010 solution terminus) to 260 Ma (base of Lopingian; Fig. 6). The Shangsi 405-kyr cycles are in phase with La2010d, shortening slightly down-section. La2010d was fitted to the INPOP06 ephemeris (INPOP, Intégration Numérique Planétaire de l′Observatoire de Paris); the other La2010 solutions were fitted to the INPOP08 ephemeris^28^. New La2011 solutions fitted to the newest ephemeris INPOP10a indicate that INPOP08 is not as good as INPOP06, the latter comparing more favourably with INPOP10a^29^. This suggests that La2010d is the most reliable of the La2010 solutions. The agreement of the U–Pb age-anchored Shangsi 405-kyr cycles to La2010d may signal that INPOP06 (and by extension INPOP10a) predicts 405-kyr eccentricity cycles accurately back to 260 Ma. In contrast, the Meishan 405-kyr cycles are not in phase with any of the solutions.

Following the widely held hypothesis of synchronous end-Permian mass extinctions^12^,^23^, we shift the Meishan series forward by 134 kyr to agree with the Shangsi chronology and La2010d solution (Fig. 1d). The adjusted 405-kyr-tuned ages remain within the U–Pb age uncertainties (cf. Fig. 1c,d).

At Meishan, this adjusted ATS indicates a PTB age of 252.10 Ma, a Wuchiapingian/Changhsingian boundary (WCB) age of 253.97 Ma and a duration of 1,870 kyr for the Changhsingian stage (Fig. 1d; Supplementary Fig. S6d; Supplementary Table S2). The Shangsi ATS gives ages of 252.10 and 254.115 Ma for the Upper and Lower Changhsingian stage boundaries and a duration of 2,015 kyr (Fig. 2c, Supplementary Fig. S7c, Supplementary Table S2).

The ages and durations of the conodont zones in the Meishan and Shangsi sections are listed in Supplementary Table S2. Conodont zones are traditionally regarded as the best correlation tool available for late Permian stratigraphy. However, according to the ATS, the ages and durations of the five Changhsingian conodont zones (*Clarkina meishanensis, C. yini, C. changxingensis, C. subcarinata* and *C. wangi*) at Meishan and Shangsi differ significantly. These differences suggest that the conodont zones could be diachronous or have uncertain boundaries.

At Meishan, the ATS gives a duration of 160 kyr for the *δ*^13^C_carb_ decline from the base of bed 23 to upper bed 24 and 15 kyr for the negative *δ*^13^C_carb_ excursion from upper bed 24 to bed 25 (Supplementary Figs S2 and S3). The maximum extinction interval (MEI) from upper bed 24 to bed 28 has a U–Pb duration of ≤200±100 kyr^12^; our ATS indicates 112 kyr. According to ref. 17, the MEI starts at the base of bed 25 and ends at the top of bed 28, for which our ATS indicates 83 kyr, much shorter than the previously estimated 700 kyr^17^. However, at Shangsi, the MEI (base of bed 27 to middle of bed 28d) has an estimated duration of 692 kyr^17^; our ATS gives 380 kyr (Supplementary Fig. S5). Our MEI duration estimate at Shangsi is bracketed by that of the previous cyclostratigraphic interpretation^17^ (692 kyr) and the U–Pb ages^12^ (200 kyr). The short MEI duration estimated at Meishan can be attributed to stratigraphic condensation/hiatus^30^.

The exceptional 7.793 Myr duration of the Shangsi ARM series provides an opportunity to seek long-period modulation patterns in the interpreted eccentricity and obliquity cycles that might be related to Earth–Mars orbital perihelion *g*_4_–*g*_3_ and inclination *s*_4_–*s*_3_ interactions. The *g*_4_–*g*_3_ term affects the amplitude of the 405-kyr eccentricity cycle, and the *s*_4_–*s*_3_ term affects the amplitude of the main 34-kyr obliquity cycle. The AM analysis (Fig. 4) reveals AM periodicity in the 405-kyr eccentricity and 34-kyr obliquity that is most similar to that of La2010d^11^ (Supplementary Fig. S15). This is consistent with the agreement of 405-kyr cycle phasing between Shangsi and La2010d, as discussed previously.

In summary, high-definition Milankovitch cycles have been discovered in well-dated Upper Permian strata in South China. Dominant 405-kyr cycles in rock magnetic data from ~1350, km-separated sedimentary sections at Meishan and Shangsi are comparable and consistent with U–Pb geochronology. One U–Pb age, 252.28 Ma, common to two dated sections, was used to anchor the cyclicity to absolute time. The 405-kyr tuning resulted in ATS ages that are consistent with the other U–Pb dates. The Meishan ATS was adjusted forward by 134 kyr to synchronize the mass extinctions recorded in the Meishan and Shangsi sections, and to phase-lock the 405-kyr cycles between the two sections. The results of this small adjustment are still consistent with the U–Pb dating. These Milankovitch cycles form the basis for a late Permian ATS that remains faithful to the U–Pb dating and indicates a 7.793-Myr duration for the Lopingian epoch. Finally, long-period modulations captured by the interpreted 405-kyr eccentricity and 34-kyr obliquity terms at Shangsi provide an opportunity to evaluate Earth–Mars resonance according to the ratio of secular frequencies of the two planets, *g*_4_–*g*_3_ and *s*_4_–*s*_3_. This evidence, together with the 405-kyr cycles, is modelled most closely by the astronomical solution La2010d. These results are an important first step towards constraining a late Permian astronomical solution and extending the ATS into the Palaeozoic era.

## Methods

### Meishan section

The Meishan section (31°4′ 55′′ N, 119°42′ 22.9′′ E) is located in Changxing County, Zhejiang Province, South China (Supplementary Fig. S1). It is the stratotype section for the Changhsingian stage with the Global Stratotype Section and Point (GSSP) for the PTB and the WCB^13^,^14^. Geologically, this section is located on the western limb of the Meishan anticline that consists of Upper Palaeozoic and Lower Triassic rocks^14^. The stratigraphic succession is well exposed and structurally simple.

The Meishan section is composed of three lithostratigraphic units including, in ascending stratigraphic order, the Longtan, Changxing and Yinkeng formations (Supplementary Figs S2 and S3). Bed 1 in this section is the uppermost of the Longtan Formation and consists of a dark-colour, dolomitized calcirudite with fragments of limestone, siltstone and phosphate. The Changxing Formation (beds 2–24; uppermost Wuchiapingian stage to Changhsingian stage) is represented by siliceous bioclastic lime mudstone with thinly interbedded cherts. The Yinkeng Formation (beds 25–29; uppermost Changhsingian to lowest Induan) consists of mainly parallel-laminated calcareous shales with minor intercalations of thin-bedded limestones. The Changxing Formation was deposited from carbonate platform/slope environment, whereas the Yinkeng Formation was deposited in an intraplatform depression dominated by shallow-water carbonates^13^,^14^. The stratigraphic contacts of Longtan/Changxing and Changxing/Yinkeng formations are conformable (Supplementary Figs S2 and S3).

A detailed conodont biostratigraphy of the Meishan D section has been established^12^,^13^,^14^,^31^. A total of nine conodont zones were identified in our studied interval (beds 1–29). In ascending order, they are: *C. orientalis* (beds 1–4a1), *C. wangi* (beds 4a2–10), *C. subcarinata* (beds 9–12), *C. changxingensis* (beds 10–24), *C. yini* (beds 24–26), *C. meishanensis* (beds 27a-b), *Hindeodus parvus* (bed 27c), *Isarcicella staeschei* (beds 27d-28), *I. isarcica* (bed 29 and above; Fig. 1, Supplementary Figs S2,S3 and S6, Supplementary Table S2). The GSSP for the PTB and the WCB was defined at the first occurrence (FO) of *H. parvus* and *C. wangi,* respectively^13^,^14^.

At the Meishan section, *δ*^13^C_carb_ of the Changxing Formation, show relatively stable values between ~3.5 and 4.2‰, a gradual decline at the base of bed 23, and then sharp negative spikes to −3.23‰ at bed 24e and bed 25 (ref. 32), followed by a sharp increase in the lower bed 26 (ref. 12; Supplementary Figs S2 and S3).

Jin *et al.* 33 summarized the magnetostratigraphic sequence. Beds 1–29 are composed of six normal polarity zones and five reversed polarity zones (Supplementary Fig. S2). The WCB is within the basal normal polarity zone, whereas the PTB is within the uppermost normal polarity zone.

Six high-precision U–Pb ID-TIMS ages of 252.10±0.06 Ma (bed 28), 252.28±0.08 Ma (bed 25), 252.50±0.11 Ma (bed 22), 252.85±0.11 Ma (bed 15), 253.45±0.08 Ma (bed 7) and 253.49±0.07 Ma (bed 6) were recently obtained at the Meishan section^12^ (Fig. 1, Supplementary Figs S2,S3 and S6). These ages are consistent with previously published ages^18^,^34^ within error, except for the ages of beds 25 and 28, which are older than those in ref. 34. The new ages^12^ are two to three times more precise than previous results. We used these new high-resolution ages to establish an initial age time framework through linear interpolation and to test the validity of our astronomical tuning^12^.

### Shangsi section

The Shangsi section (32°20′ N, 105°28′ E) is located in the town of Shangsi, Guangyuan City, Sichuan Province, southwest China. It was formerly one of the GSSP candidate sections for the PTB (ref. 35). Geologically, this section is situated at the southeast limb of an anticline that belongs to the southern flank of the Indosinian Longmenshan fold zone, which is part of the Qinling fold belt along the northern margin of the South China block^35^,^36^.

The section is well exposed in a road cut and in a parallel section along a river. Carbonate-dominated marine strata of the Wujiaping, Dalong and Feixianguan formations span the Lower Wuchiapingian stage to Changhsingian stage of the Lopingian (Upper Permian) and the Induan stage (Lower Triassic)^25^,^37^ (Supplementary Figs S4 and S5). The Wujiaping Formation (beds 5–10) consists primarily of thick-bedded limestone with chert nodules and was deposited from carbonate-dominated, neritic shelf environments^15^. The conformably overlying Dalong Formation (beds 11–27) is composed of medium-bedded bioturbated siliceous limestone, interbedded with thinly bedded organic-rich limestone and shale, with total organic carbon (TOC) content up to 14% (ref. 38). The depositional environments of the Dalong Formation evolved from platform margin/upper slope, to outer shelf, and to intrashelf basin^15^. The Feixianguan Formation (bed 28 and above) consists of finely laminated micritic limestone, marl and organic-rich shale^25^,^35^. The lithologic boundary between the Dalong and Feixianguan formations marks a disruption of carbonate deposition and increase of siltstones and mudstones (Supplementary Figs S4 and S5). The hemipelagic facies in the Dalong and Feixianguan formations in Shangsi indicates a deeper-water depositional environment in comparison with the Meishan section^13^,^39^.

The Shangsi section has undergone detailed biostratigraphic investigation^12^,^35^,^39^,^40^,^41^. In ascending order, nine established conodont zones are: *C. transcaucasica* (beds 11–lower bed 18); *C. orientalis* (bed 16–lower bed 18); *C. wangi* (lower bed 18–base bed 19); *C. subcarinata* (upper bed 18–bed 19); *C. changxingensis* (beds 20–27); *C. yini* (bed 26); *C. meishanensis* (beds 27–28a); *H. eurypyge* (beds 28b–29); *H. parvus* zone (bed 29c and above)^12^,^41^ (Supplementary Figs S4,S5 and S7).

The Changhsingian conodont zones are consistent with those of the Meishan section^12^ (Figs 1 and 2, Supplementary Figs S2–S7). The Wuchiapingian/Changhsingian stage boundary was placed at the base of *C. wangi* zone. However, the FO *H. parvus,* which is regarded as the base of the Triassic in the Meishan section^13^, was identified at strata 2.0 m (ref. 41) or 4.5 m (refs 39, 40) above the Dalong/Feixianguan formation boundary. The PTB was placed at the bed 28b/28c boundary (0.4 m above the Dalong/Feixianguan formation boundary) based on FO *H. eurypyge* (12) or other index conodont fossils such as *H. changxingensis* and *Neogondoelella taylorae*, which are 0.22 m above the base of bed 28 (ref. 41). Here we adopted the zonation used by Shen *et al.*^12^

The *δ*^13^C_carb_ values show a sharp negative excursion at beds 25–29, which is similar to that at Meishan and other sections^16^,^42^ (Supplementary Figs S4 and S5). A composite magnetostratigraphy based on three sections (beds 5–48) from the Shangsi area^36^ shows at least eight polarity chrons, and the PTB occurs within a normal polarity chron, 50 cm above the mass extinction level (Supplementary Figs S4 and S5).

Eight high-precision, single zircon U–Pb ID-TIMS ages were obtained from the Shangsi section^12^ (Fig. 2, Supplementary Figs S4,S5 and S7). These include (1) 252.16±0.09 Ma, 50 cm above the extinction horizon (bed 27/28 boundary or 10 cm above the PTB); (2) 252.28±0.13 Ma, 20 cm below the bed 27/28 boundary; and (3) 252.37±0.08 Ma, 30 cm below the bed 27/28 boundary. These ages indicate that the mass extinction interval occurred during 252.37–252.16 Ma, which is consistent with the age estimate from the Meishan section^12^. Ages at 1, 2.9, 12.68, 17.1 and 27.5 m below the bed 27/28 boundary are 252.68±0.12, 253.10±0.12, 253.60±0.08, 254.31±0.07 and 257.79±0.14 Ma, respectively^12^ (Supplementary Figs S4,S5 and S7). The base of the Wuchiapingian stage was estimated as 259–260 Ma (refs 43 and 44). We used the age of 259.5±0.9 Ma from upper bed 6 (ref. 18) and ages from ref. 12 to construct the initial age framework.

### Rock magnetic time series

High-resolution time series of MS and ARM were obtained from the Meishan and Shangsi sections to search for Milankovitch sedimentary cycles. The MS is a measure of the degree of magnetization of a material in response to an applied magnetic field. Many studies have demonstrated that MS can be a powerful tool for cyclostratigraphy and paleoclimate studies^17^,^45^,^46^,^47^. Recent studies suggest that ARM may be a better proxy for cyclostratigraphic study of sedimentary successions because ARM measures the concentration of fine-grained, low-coercivity ferromagnetic minerals that have a relatively simpler origin than MS^47^,^48^,^49^.

The Meishan section has been protected from collecting samples as it was ratified by the International Union of Geological Sciences as the GSSP for the PTB in 2001 (ref. 13). Therefore, we used a portable MS meter, SM30 with sensitivity of 10^–7^ SI, to measure MS every 2 cm at the outcrop. A total of 1,970 measurements were conducted.

At the Shangsi section, we collected 2,700 specimens at a spacing of 5 cm in the Wujiaping Formation and 1–2 cm in the Dalong and Feixianguan formations. Weathered, fractured and diagenetically altered zones were avoided by laterally tracing the beds into better exposures. All specimens were crushed and put into 8-cm^3^ non-magnetic cubic plastic boxes. The ARM was acquired by applying a peak-alternating field of 0.1 T and a bias field of 50 μT on a D-2000 AF demagnetizer. ARM remanence intensity measurements were made on a JR6 spinner magnetometer. Sample processing and measurements were conducted in the Paleomagnetism and Environmental Magnetism Laboratory at China University of Geosciences (Beijing).

MS values in the Meishan section range from −1.4 × 10^−5^ SI to 7.4 × 10^−5^ SI with an average value of 1.98 × 10^−5^ SI (Fig. 1, Supplementary Figs S2,S3 and S6). ARM values in the Shangsi section range from 0.025 to 5.67 × 10^−6^ Am^2^ kg^−1^ with the average value of 0.78 × 10^−6^ Am^2^ kg^−1^ (Fig. 2, Supplementary Figs S4,S5 and S7). Both ARM and MS show relatively stable values in the Upper Permian with clear short-period variations superimposed on long-period fluctuations.

The variations in the MS and ARM series closely track lithological changes, that is, higher values correspond to marls, mudstone or shales, whereas lower values correspond to limestone (Supplementary Figs S2–S5). Magnetic experiments with samples from the Shangsi section indicate that the main magnetic minerals are low-coercivity titanomagnetite and the natural remanent magnetization intensities of limestone samples are lower than those of the marls, muddy limestones and mudstones^36^. Therefore, the ARM and MS fluctuations most probably reflect variations in the ratio of terrestrial siliciclastics (that is, detrital magnetic material input) to marine carbonate.

We propose that astronomically forced climate change influenced the MS and ARM variations and that both have the same response to climate change with same phase. During times of high eccentricity, climate change induced by precession results in higher precipitation, higher continental runoff and, ultimately, higher sedimentary MS and ARM.

The phase relationship between the MS (or ARM) and eccentricity is also supported by lithological changes (Supplementary Figs S5 and S11). The lithological changes in beds 19–28 show that precession-scale layers with higher muddy content have more distinct bed boundaries during eccentricity maxima, whereas weak precession-scale layers are dominated by limestone during eccentricity minima.

### Time-series methods

The MS and ARM stratigraphic series were linearly interpolated to a uniform spacing of 1 cm and resampled in Analyseries 2.0.4.2 (ref. 50), and then pre-whitened before spectral analysis by removing 40% (MS) and 35% (ARM) weighted averages with KaleidaGraph^51^. The MS and ARM time series in the U–Pb age initial time framework and 405-kyr-tuned time framework were linearly interpolated and resampled to a uniform spacing of 1 kyr (Shangsi) and 0.2 kyr (Meishan), and pre-whitened by removing a 35% weighted average (Shangsi) and a 66% weighted average (Meishan).

Multitaper method (MTM) spectral analysis^52^, evolutionary fast Fourier transform spectrograms and wavelet analysis^53^ were conducted on the MS and ARM series to identify the sedimentary cycles. The cycle length ratio method^54^ was applied to investigate links between detected sedimentary cycles and astronomical forcing. MTM power spectral analysis was conducted using the SSA-MTM toolkit^55^ downloaded from the website: http://www.atmos.ucla.edu/tcd/ssa/. Robust estimation of background red noise with confidence limits at 90, 95 and 99% level was determined following ref. 56. Wavelet analysis software^53^ was downloaded from http://www.paos.colorado.edu/research/wavelets.

The interpreted 405-kyr eccentricity and 34-kyr obliquity cycles were extracted with Gaussian band-pass filters in Analyseries 2.0.4.2 (ref. 50) and Taner band-pass filters^57^ in Matlab. AM envelopes of the filtered series were obtained using Hilbert transformation.

The identification of Milankovitch cycles was based on the high-precision U–Pb age framework^12^. The MS and ARM data were also tuned to the interpreted 405-kyr eccentricity cycles and anchored to the U–Pb age of 252.28 Ma that was obtained in both sections. The U–Pb ages and 405 kyr-tuned MS and ARM series were used jointly to assess the presence and periods of short (~100-kyr scale) eccentricity, obliquity and precession cycles. The tuning process was conducted in Analyseries 2.0.4.2 (ref. 50).

## Author contributions

H.W. designed the study, collected samples and conducted experiments. H.W. and L.A.H. wrote the paper. All authors contributed to the interpretation of the data and provided significant input to the final manuscript.

## Additional information

**How to cite this article:** Wu, H. *et al.* Time-calibrated Milankovitch cycles for the late Permian. *Nat. Commun.* 4:2452 doi: 10.1038/ncomms3452 (2013).

## Supplementary Material

Supplementary Information Supplementary Figures S1-S15 and Supplementary Tables S1-S2

## References

[b1] Berger A. L. & Loutre M. F. Astronomical theory of climate change. J. Phys. IV France 121, 1–35 (2004).

[b2] Shackleton N. J., McCave N. & Weedon G. P. Astronomical (Milankovitch) calibration of the geological time–scale: a discussion. Philos. Trans. R. Soc. Lond. Ser. A 357, 1733–2007 (1999).

[b3] D′Argenio B., Fischer A. G., Premoli Silva I., Weissert H. & Ferreri V. Cyclostratigraphy: approaches and case histories. SEPM Special Publication 81, 1–311 (2004).

[b4] Olsen P. E. & Whiteside J. H. inEncyclopedia of Paleoclimatology and Ancient Environments eds Gornitz V. 826–835Springer Verlag (2009).

[b5] Pälike H. & Hilgen F. Rock clock synchronization. Nat. Geosci. 1, 282–282 (2008).

[b6] Hinnov L. A. & Hilgen F. inA Geologic Time Scale 2012 eds Gradstein F., Ogg J., Ogg G., Smith D. 63–83Elsevier (2012).

[b7] Kuiper K. F. *et al.* Synchronizing rock clocks of Earth history. Science 320, 500–504 (2008). 18436783 10.1126/science.1154339

[b8] Rivera T. A., Storey M., Zeeden C., Hilgen F. & Kuiper K. A refined astronomically calibrated ^40^Ar/^39^Ar age for the Fish Canyon Sanidine. Earth Planet. Sci. Lett. 311, 420–426 (2011).

[b9] Meyers S. *et al.* Intercalibration of radioisotopic and astrochronologic time scales for the Cenomanian-Turonian Boundary interval, Western Interior Basin, USA. Geology 40, 7–10 (2012).

[b10] Laskar J. *et al.* A long term numerical solution for the insolation quantities of the Earth. Astron. Astrophys. 428, 261–285 (2004).

[b11] Laskar J., Fienga A., Gastineau M. & Manche H. La2010: a new orbital solution for the long-term motion of the Earth. Astron. Astrophys. 532, A89 (2011).

[b12] Shen S. Z. *et al.* Calibrating the end-Permian mass extinction. Science 334, 1367–1372 (2011). 22096103 10.1126/science.1213454

[b13] Yin H. F., Zhang K. X., Tong J. N., Yang Z. Y. & Wu S. B. The Global Stratotype Section and Point (GSSP) of the Permian–Triassic boundary. Episodes 24, 102–114 (2001).

[b14] Jin Y. G. *et al.* The Global Stratotype Section and Point (GSSP) for the base-Changhsingian stage (upper Permian). Episodes 29, 175–182 (2006).

[b15] Yan J. X. *et al.* Subdivision of Permian fossil communities and habitat types in Northeast Sichuan, South China. J. China Univ. Geosci. 19, 441–450 (2008).

[b16] Cao C. Q. *et al.* Pattern of δ^13^C_carb_ and implications for geological events during the Permian-Triassic transition in South China. Geol. J. 45, 186–194 (2010).

[b17] Huang C. J., Tong J. N., Hinnov L. A. & Chen Z. Q. Did the great dying take 700 k.y.? Evidence from astronomical correlation of the Permian –Triassic boundary interval. Geology 39, 779–782 (2011).

[b18] Mundil R., Ludwig K. R., Metcalfe I. & Renne P. R. Age and timing of the Permian mass extinctions: U/Pb dating of closed-system zircons. Science 305, 1760–1763 (2004). 15375264 10.1126/science.1101012

[b19] Jin Y. G. *et al.* Pattern of marine mass extinction near the Permian–Triassic boundary in South China. Science 289, 432–436 (2000). 10903200 10.1126/science.289.5478.432

[b20] Erwin D. H. Extinction: How life on Earth Nearly Ended 250 Million Years Ago 296, Princeton Univ. Press (2006).

[b21] Chen Z. Q. & Benton M. J. The timing and pattern of biotic recovery following the end-Permian mass extinction. Nat. Geosci. 5, 375–383 (2012).

[b22] Grasby S. E., Sanei H. & Beauchamp B. Catastrophic dispersion of coal fly ash into oceans during the latest Permian extinction. Nat. Geosci. 4, 104–107 (2011).

[b23] Yin H. F., Feng Q. L., Lai X. L., Baud A. & Tong J. N. The protracted Permo-Triassic crisis and multi-episode extinction around the Permian-Triassic boundary. Global Planet. Change 55, 1–20 (2007).

[b24] Algeo T. J. & Twitchett R. J. Anomalous early Triassic sediment fluxes due to elevated weathering rates and their biological consequences. Geology 38, 1023–1026 (2010).

[b25] Li Z. S. *et al.* Mass extinction and geological events between palaeozoic and Mesozoic era. Acta Geol. Sin. 60, 1–17 (1986).

[b26] Olsen P. E. & Kent D. V. Long-period Milankovitch cycles from the late Triassic and early Jurassic of eastern North America and their implications for the calibration of the early Mesozoic time-scale and the long-term behaviour of the planets. Philos. Trans. R. Soc. Lond. 357, 1761–1786 (1999).

[b27] Hinnov L. A. New perspectives on orbitally forced stratigraphy. Annu. Rev. Earth Planet. Sci. 28, 419–475 (2000).

[b28] Fienga A. *et al.* INPOP08, a 4-D planetary ephemeris: from asteroid and time-scale computations to ESA Mars express and Venus express contributions. Astron. Astrophys. 507, 1675–1686 (2009).

[b29] Westerhold T., Röhl U. & Laskar J. Time scale controversy: accurate orbital calibration of the early Paleogene. Geochem. Geophys. Geosyst. 13, Q06015 (2012).

[b30] Cao C. Q. & Zheng Q. F. Geological event sequences of the Permian-Triassic transition recorded in the microfacies in Meishan section. Sci. China Ser. D-Earth Sci. 52, 1529–1536 (2009).

[b31] Jiang H. S. *et al.* Restudy of conodont zonation and evolution across P/T Boundary at Meishan Section, Changxing, Zhejiang, China. Global Planet. Change 55, 39–55 (2007).

[b32] Cao C. Q. *et al.* Biogeochemical evidence for euxinic oceans and ecological disturbance presaging the end-Permian mass extinction event. Earth Planet. Sci. Lett 281, 188–201 (2009).

[b33] Jin Y. G., Shang Q. H. & Cao C. Q. Late Permian magnetostratigraphy and its global correlation. Chin. Sci. Bull. 45, 668–700 (2000).

[b34] Bowring S. A. *et al.* U/Pb zircon geochronology and tempo of the end-Permian mass extinction. Science 280, 1039–1045 (1998). 9582110 10.1126/science.280.5366.1039

[b35] Lai X. L., Yang F. Q., Hallam A. & Wignall P. B. inThe Palaeozoic-Mesozoic Boundary candidates of Global Stratotype Section and Point of the Permian-Triassic Boundary ed Yin H. F. 113–124China Univ. Geoscience Press (1996).

[b36] Glen J. M. G. *et al.* Magnetostratigraphic correlations of Permian–Triassic marine-to-terrestrial sections from China. J. Asian Earth Sci. 36, 521–540 (2009).

[b37] Wignall P. B., Hallam A., Lai X. & Yang F. Palaeoenvironmental changes across the Permian-Triassic boundary at Shangsi (N. Sichuan, China). Hist. Biol. 10, 175–189 (1995).

[b38] Chen H., Xie X. N., Hu C. Y., Huang J. H. & Li H. J. Geochemical characteristics of Late Permian sediments in the Dalong Formation of the Shangsi Section, Northwest Sichuan Basin in South China: implications for organic carbon-rich siliceous rocks formation. J. Geochem. Exp. 112, 35–53 (2012).

[b39] Nicoll R. S., Metcalfe I. & Wang C. Y. New species of the conodont Genus Hindeodus and the conodont biostratigraphy of the Permian-Triassic boundary interval. J. Asian Earth Sci. 20, 609–631 (2002).

[b40] Metcalfe I., Nicoll R. S. & Wardlaw B. R. Conodont index fossil *Hindeodus changxingensis* Wang fingers greatest mass extinction event. Paleoworld 16, 202–207 (2007).

[b41] Jiang H. S. *et al.* Revised conodont zonation and conodont evolution across the Permian–Triassic boundary at the Shangsi section, Guangyuan, Sichuan, South China. Global Planet. Change 72, 103–115 (2011).

[b42] Tong J. N., Zuo J. X. & Chen Z. Q. Early Triassic carbon isotope excursions from South China: proxies for devastation and restoration of marine ecosystems following the end-Permian mass extinction. Geol. J. 42, 371–389 (2007).

[b43] Shen S. Z. *et al.* High-resolution Lopingian (Late Permian) timescale of South China. Geol. J. 45, 122–134 (2010).

[b44] Gradstein F. M., Ogg J. G., Schmitz M. & Ogg G. The Geologic Time Scale 2012 Elsevier (2012).

[b45] Boulila S. *et al.* Milankovitch and sub-Milankovitch forcing of the Oxfordian (Late Jurassic) Terres Noires Formation (SE France) and global implications. Basin Res. 22, 717–732 (2010).

[b46] Wu H. C. *et al.* Astrochronology of the Early Turonian–Early Campanian terrestrial succession in the Songliao Basin, northeastern China and its implication for long-period behavior of the Solar System. Palaeogeogr. Palaeoclimatol. Palaeoecol. 385, 55–70 (2013).

[b47] Wu H. C. *et al.* Milankovitch and sub-Milankovitch cycles of the Early Triassic Daye Formation, South China and their geochronological and paleoclimatic implications. Gondwana Res. 22, 748–759 (2012).

[b48] Latta D. K., Anastasio D. J., Hinnov L. A., Elrick M. & Kodama K. P. Magnetic record of Milankovitch rhythms in lithologically non-cyclic marine carbonates. Geology 34, 29–32 (2006).

[b49] Kodama K. P., Anastasio D. J., Newton M. L., Pares J. M. & Hinnov L. A. High-resolution rockmagnetic cyclostratigraphy in an Eocene flysch, Spanish Pyrenees. Geochem. Geophys. Geosyst. 11, Q0AA07 (2010).

[b50] Paillard D., Labeyrie L. & Yiou P. Macintosh program performs time-series analysis. Eos 77, 379–379 (1996).

[b51] Cleveland W. S. Robust locally weighted regression and smoothing scatterplots. J. Am. Stat. Assoc. 74, 829–836 (1979).

[b52] Thomson D. J. Spectrum estimation and harmonic analysis. Proc. IEEE 70, 1055–1096 (1982).

[b53] Torrence C. & Compo G. P. A practical guide to wavelet analysis. Bull. Am. Meteorol. Soc. 79, 61–78 (1998).

[b54] Weedon G. Time-Series Analysis and Cyclostratigraphy 1–259Cambridge Univ. Press (2003).

[b55] Ghil M. *et al.* Advanced spectral methods for climatic time series. Rev. Geophys. 40, 1–41 (2002).

[b56] Mann M. E. & Lees J. M. Robust estimation of background noise and signal detection in climatic time series. Clim. Change 33, 409–445 (1996).

[b57] Taner M. T. InAttributes Revisited Technical Publication, Rock Solid Images, Inc. (2000).

