# Supplementary material for: Time-calibrated Milankovitch cycles for the late Permian
Source: Nat Commun. 2013 Sep 13;4:2452. doi: 10.1038/ncomms3452 (PMC3778519; doi:10.1038/ncomms3452)
Supplement: Supplementary Information — Supplementary Figures S1-S15 and Supplementary Tables S1-S2 [file ncomms3452-s1.pdf]

SUPPLEMENTARY INFORMATION

Supplementary Figures

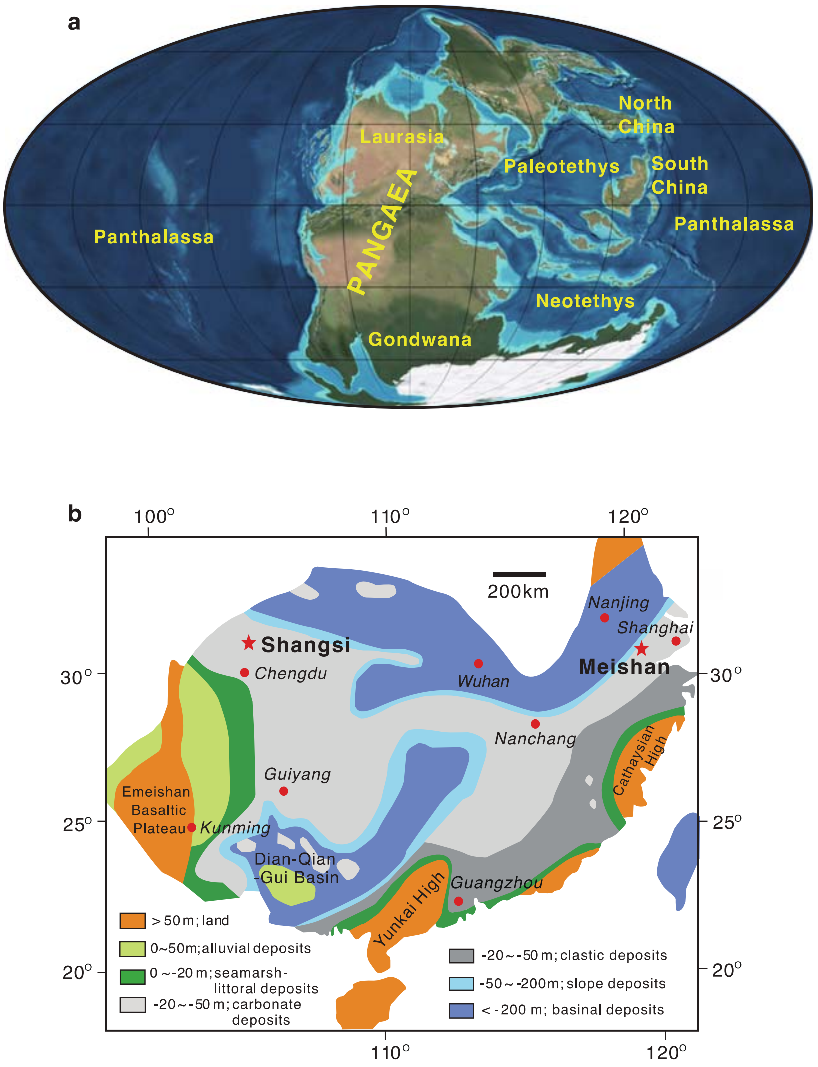

Supplementary Figure S1 | Late Permian paleogeography. (**a**) Late Permian (~260 Ma) global paleogeography showing the location of South China (base map is from Ron Blakey, http://jan.ucc.nau.edu/~rcb7). (**b**) Late Permian paleogeographic configuration of South China showing the location of the Meishan and Shangsi sections (red stars) (base map after ref. 12).

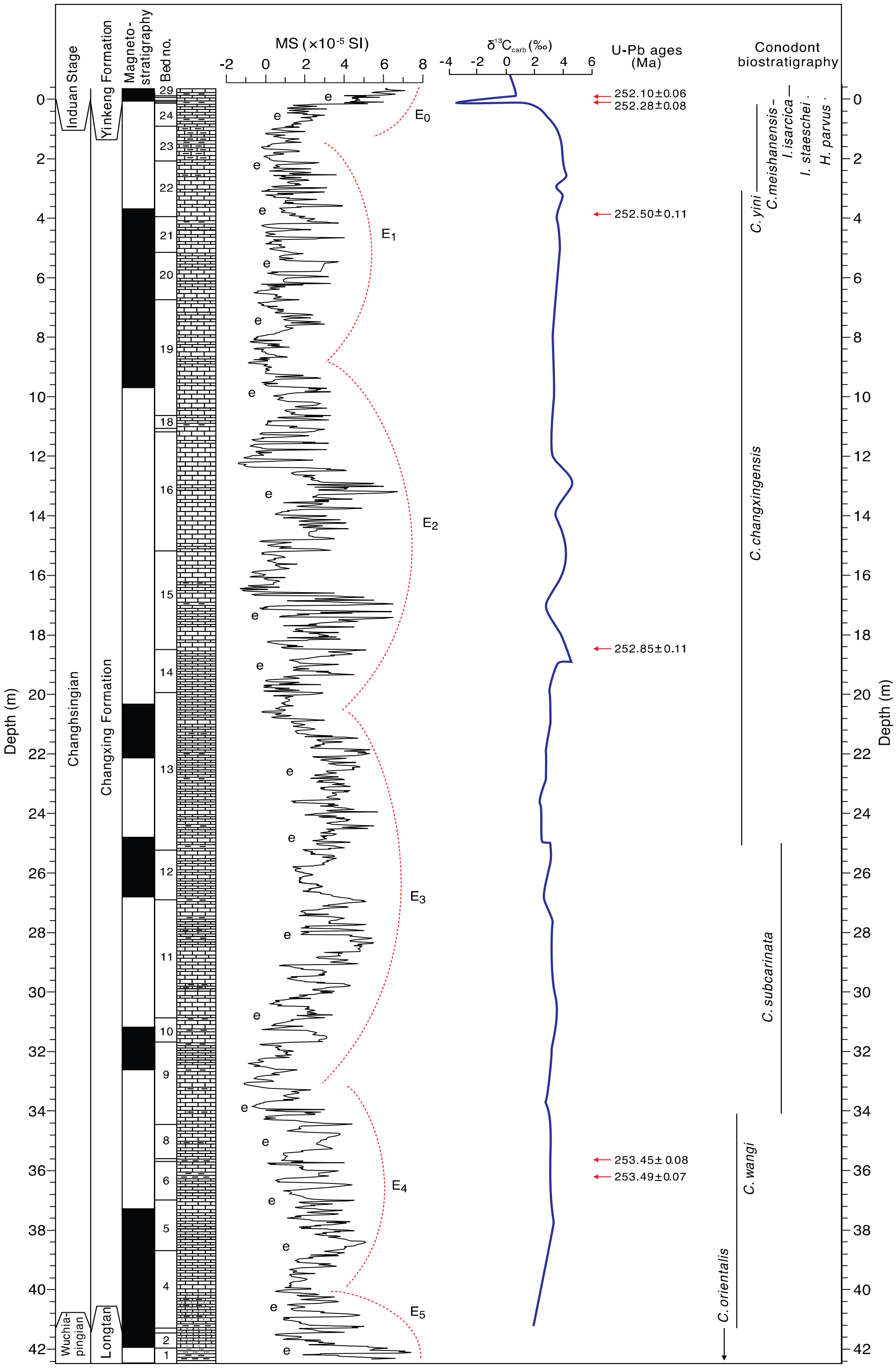

Supplementary Figure S2 | Geochronology, magnetostratigraphy, lithostratigraphy, chemostratigraphy, cyclostratigraphy and conodont biostratigraphy of the upper Longtan, Changxing, and lower Yinkeng formations at the Meishan section. Bed numbers are after refs. 13 and 14. Magnetostratigraphy is after ref. 33. Conodont biozones are from refs. 12-14 and 31. Carbon isotope data and U-Pb ages are from ref. 12. The letter 'E' and 'e' represent interpreted 405-kyr and 100-kyr eccentricity cycles in the MS series.

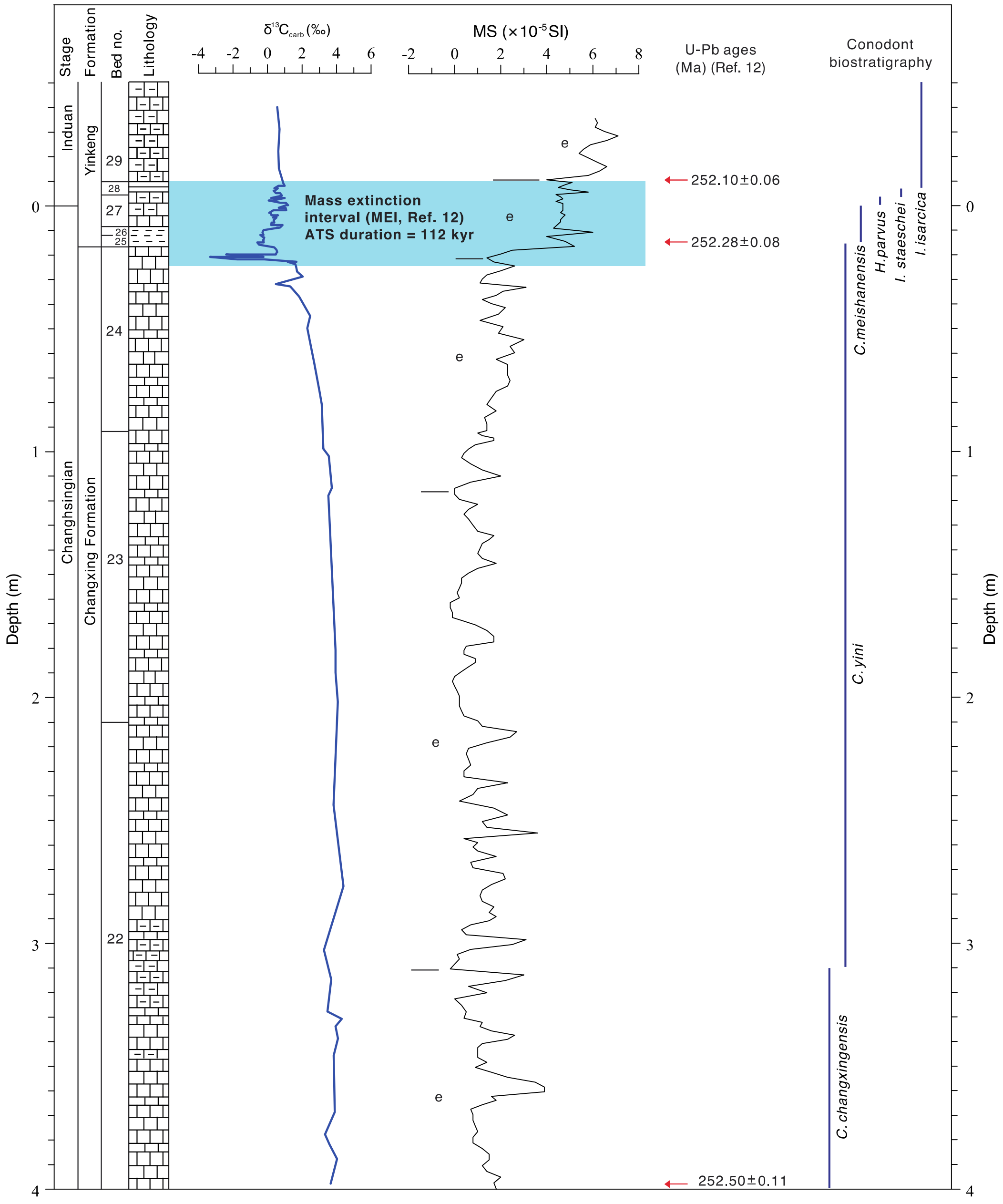

Supplementary Figure S3 | Geochronology, lithostratigraphy, chemostratigraphy, cyclostratigraphy and conodont biostratigraphy of the upper Changxing and lower Yinkeng formations at the Meishan section. Bed numbers are after ref. 13. Conodont biozones are from refs. 12, 13 and 31. Carbon isotope data and U-Pb ages are from ref. 12. The letter 'e' represents interpreted 100-kyr eccentricity cycles in the MS series. Blue shaded area indicates mass extinction interval (MEI).

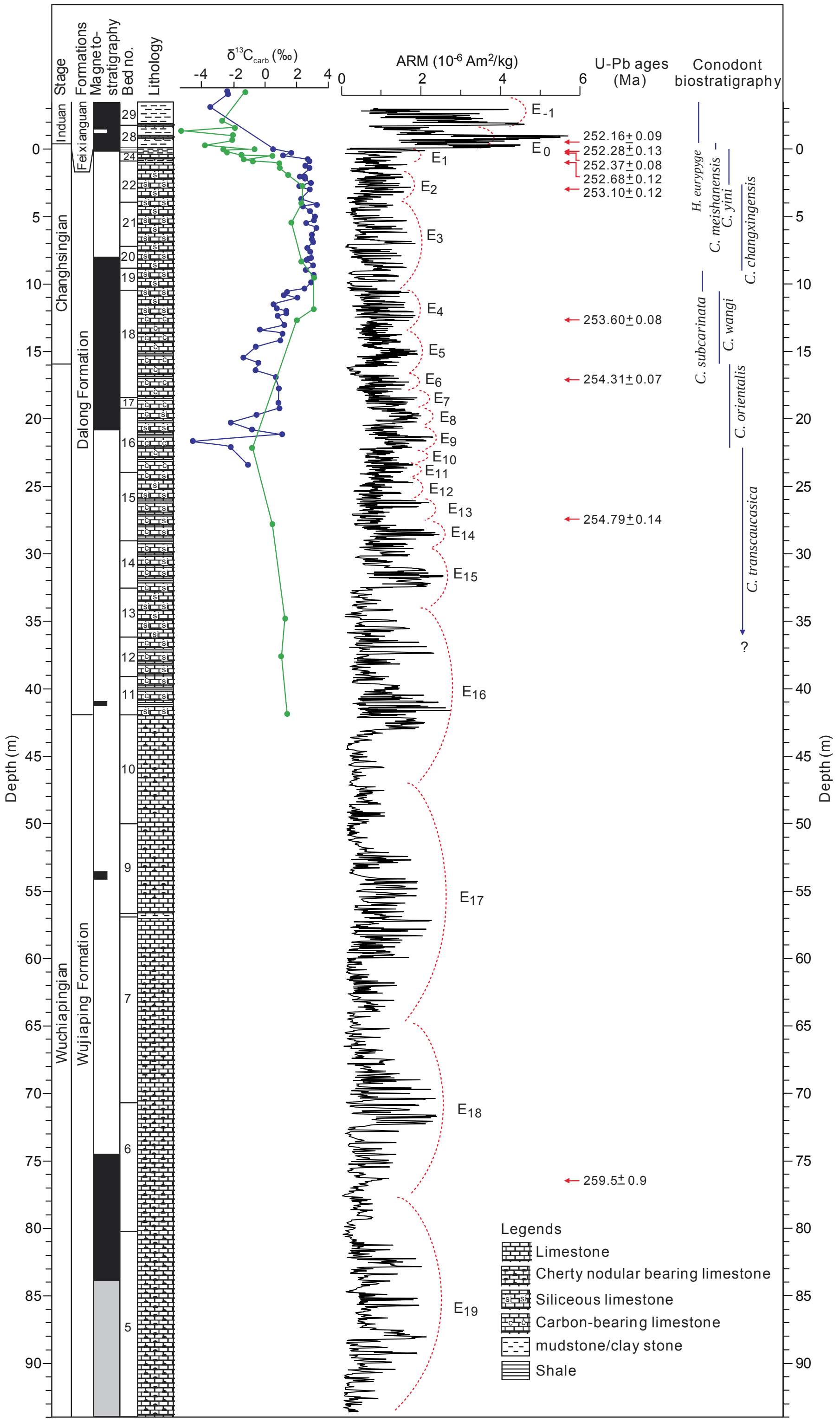

Supplementary Figure S4 | Geochronology, magnetostratigraphy, lithostratigraphy, chemostratigraphy, cyclostratigraphy and conodont biostratigraphy of the Wujiaping, Dalong, and lower Feixianguan formations at the Shangsi section. Bed numbers are after ref. 25. Magnetostratigraphy is after ref. 36. Conodont biozones and carbon isotope are from ref. 12. U-Pb ages are from refs. 12, except the age of 259.5 ± 0.9 Ma in the lower part from ref. 18. The letter 'E' represents 405-kyr eccentricity cycles in the ARM series.

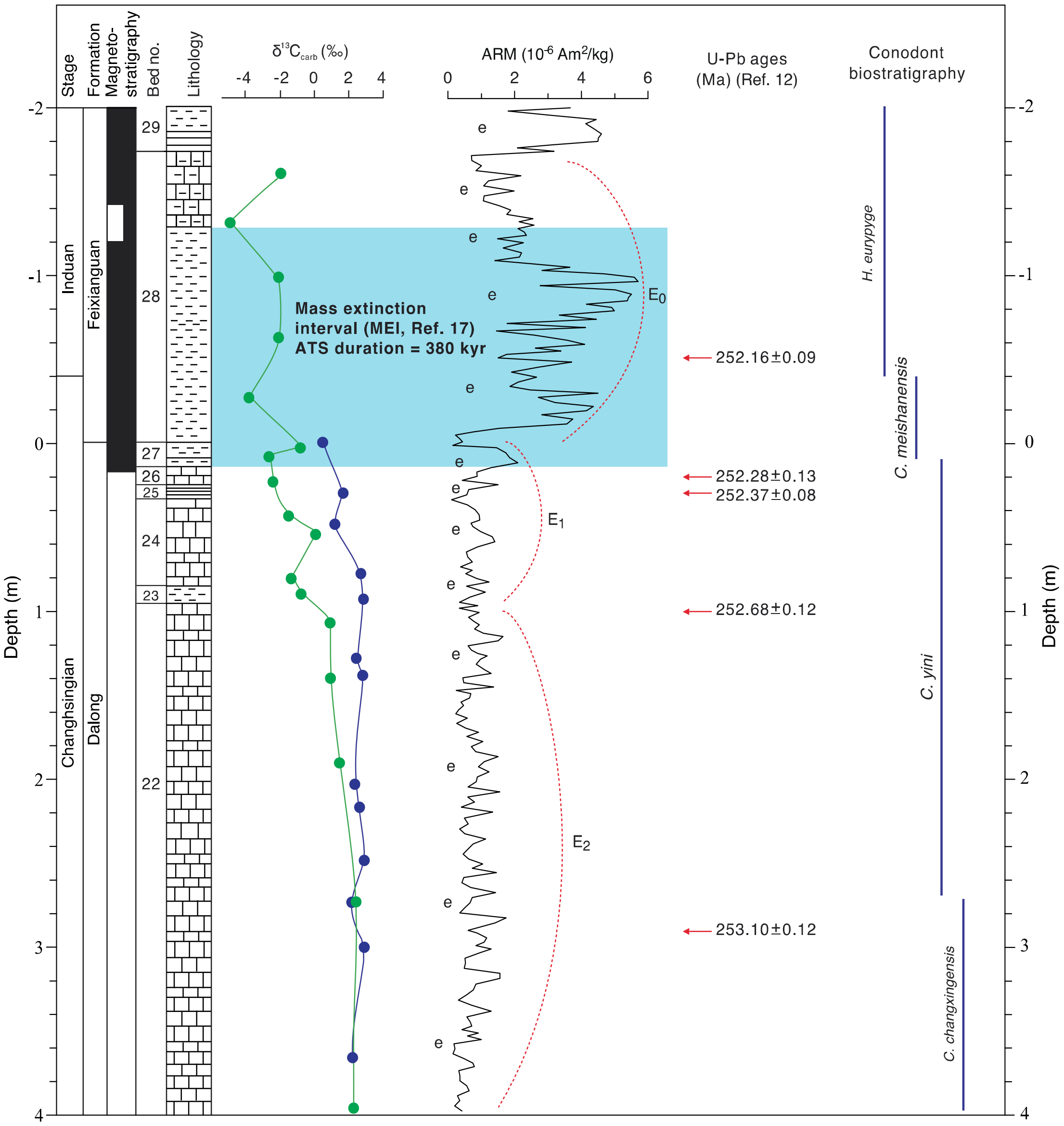

Supplementary Figure S5 | Geochronology, magnetostratigraphy, lithostratigraphy, chemostratigraphy, cyclostratigraphy and conodont biostratigraphy of upper Dalong and lower Feixianguan formations at the Shangsi section. Bed numbers are after ref. 25. Magnetostratigraphy is after ref. 36. Conodont biozones and carbon isotope are from ref. 12. U-Pb ages are from refs. 12. The letters 'E' and 'e' represent 405-kyr and 100-kyr eccentricity cycles in the ARM series. Blue shaded area indicates mass extinction interval (MEI).

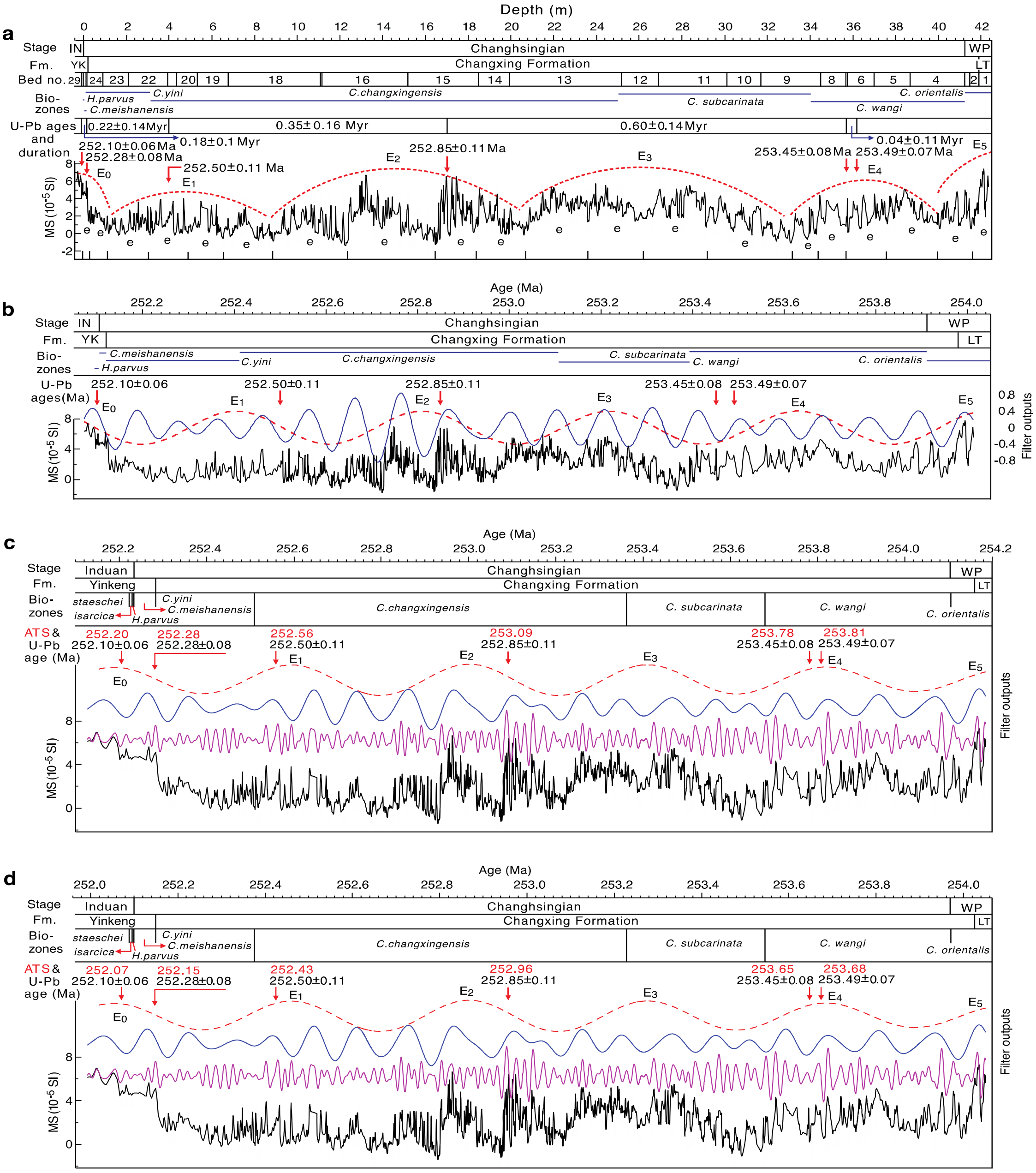

Supplementary Figure S6 | Tuning the Meishan MS stratigraphic series. (**a**) MS series in stratigraphic depth at the Meishan section. Also shown are the bed boundaries (refs. 13 and 14), conodont biozones (ref. 12), and U-Pb ages and durations (ref. 12). Uncertainties of the durations are calculated by error propagation. The interpretation of 405-kyr eccentricity (E) and ~100-kyr eccentricity (e) cycles was based on the spectral analysis in Fig. 3, Supplementary Figs. S8 and S9. (**b**) The integrated stratigraphy and cycle analysis in the time framework constrained by five U-Pb ages (ref. 12). The 405-kyr (red) and 100-kyr (blue) cycles were extracted with Gauss filters with passbands of 0.002469 ± 0.00025 cycles/kyr and 0.01 ± 0.002 cycles/kyr, respectively. (**c**) 405-kyr tuned MS series with 405-kyr (red), 100-kyr (blue) and 20-kyr (purple) filter outputs, with passbands of 0.002469 ± 0.00025 cycles/kyr, 0.01 ± 0.0035 cycles/kyr and 0.05 ± 0.025 cycles/kyr, respectively. The astronomically tuned ages (red) are shown with the U-Pb ages for comparison. (**d**) adjusted 405-kyr tuned MS time series matching the Shangsi chronology at PTB (see main text). Fm.=Formation, IN=Induan, WP=Wuchiapingian, YK=Yinkeng, LT=Longtan.

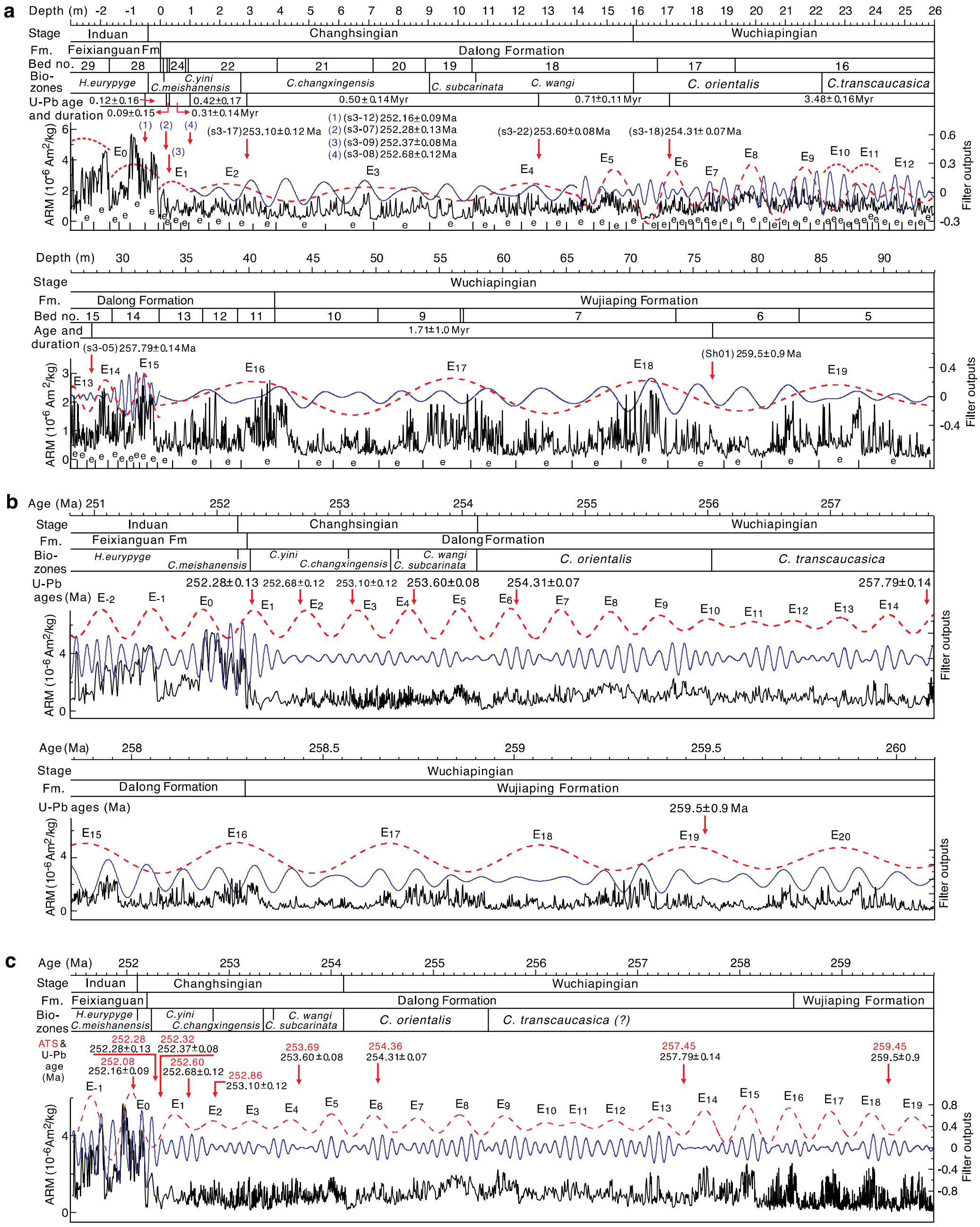

Supplementary Figure S7 | Tuning the Shangsi ARM stratigraphic series. (**a**) ARM series in depth at the Shangsi section. The interpreted 405-kyr cycles (red) were extracted with Gauss filters with passbands of 0.22 ± 0.08 cycles/m (1-14 m), 0.6 ± 0.3 cycles/m (14-26 m), 0.4 ± 0.12 cycles/m (26-33 m) and 0.06 ± 0.02 cycles/m (33-93.6 m). The 100-kyr cycles (blue) band-pass filters are 0.8 ± 0.2 cycles/m (1-14 m), 2.3 ± 0.7 cycles/m (14-26 m), 1.7 ± 0.34 cycles/m (26-33 m) and 0.26 ± 0.08 cycles/m (33-93.6 m). The U-Pb ages are from ref. 12 except the age of 259.5 ± 0.9 Ma in the lower part from ref. 18. The duration uncertainties are calculated by error propagation. Bed numbers are after ref. 25 and conodont biozones are after ref. 12. (**b**) U-Pb ages calibrated ARM series with 405-kyr (red) and 100-kyr (blue) signals extracted with Gauss filters with passbands of 0.002469 ± 0.00015 cycles/kyr and 0.01 ± 0.0015 cycles/kyr. (**c)** 405-kyr tuned ARM series with 405-kyr (red) and 100-kyr (blue) filter outputs extracted using Gauss filters with passbands of 0.002469 ± 0.00075 cycles/kyr and 001 ± 0.0025 cycles/kyr. Fm.=Formation.

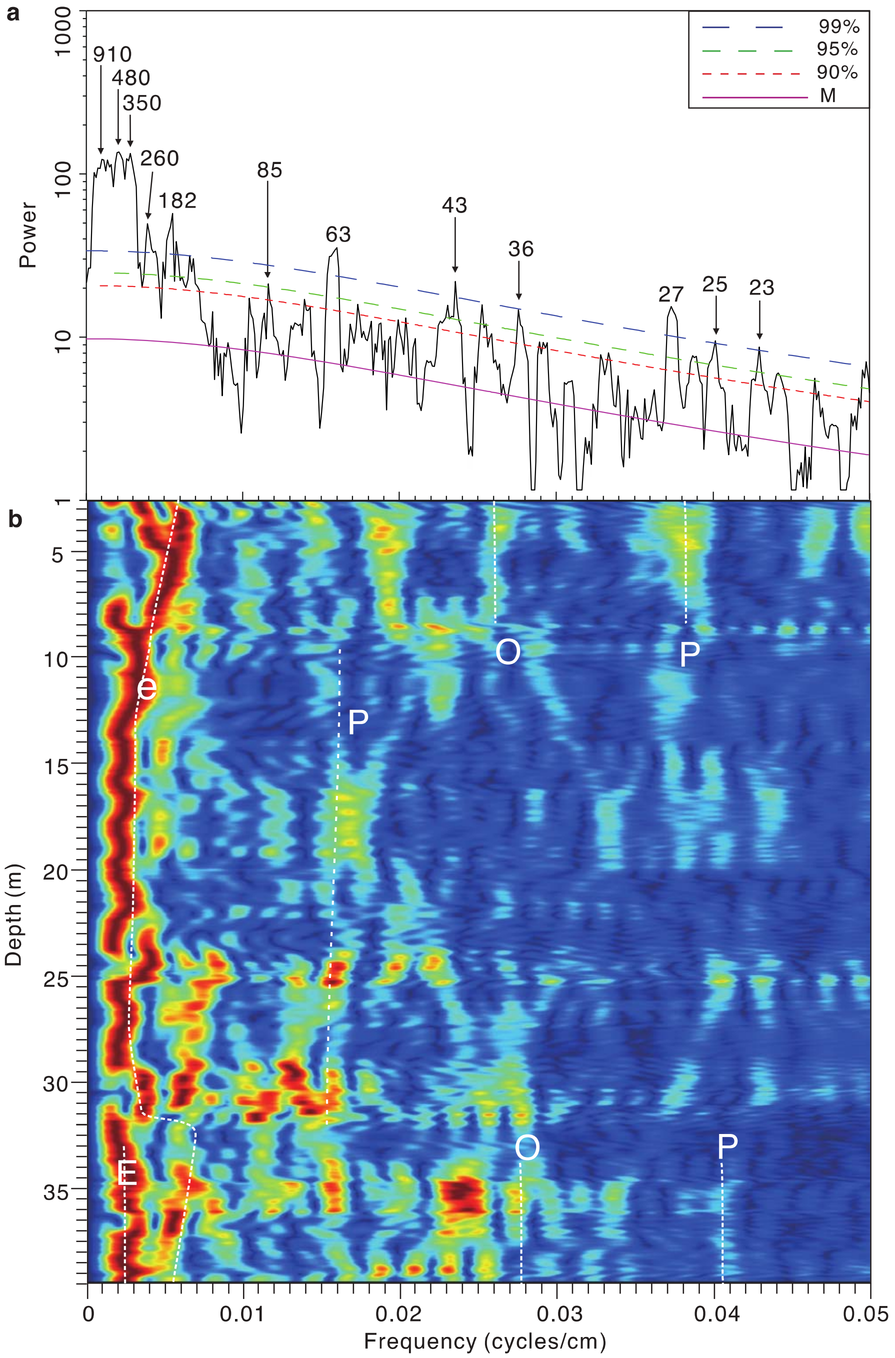

Supplementary Figure S8 | Meishan MS stratigraphic series spectrum. (**a**) 2π MTM power spectrum, with significant peaks labeled in cm. The purple, red, green and blue (dashed) curves represent the median smoothed, linear fitted red noise spectrum, and 90%, 95% and 99% confidence levels. (**b**) Evolutionary FFT spectrum, with a 6 m sliding window. The red and blue colors represent high and low power, normalized to 1. The dashed white lines labeled with E, e, O and P representing the 405-kyr eccentricity, 100-kyr eccentricity, obliquity and precession cycles, respectively.

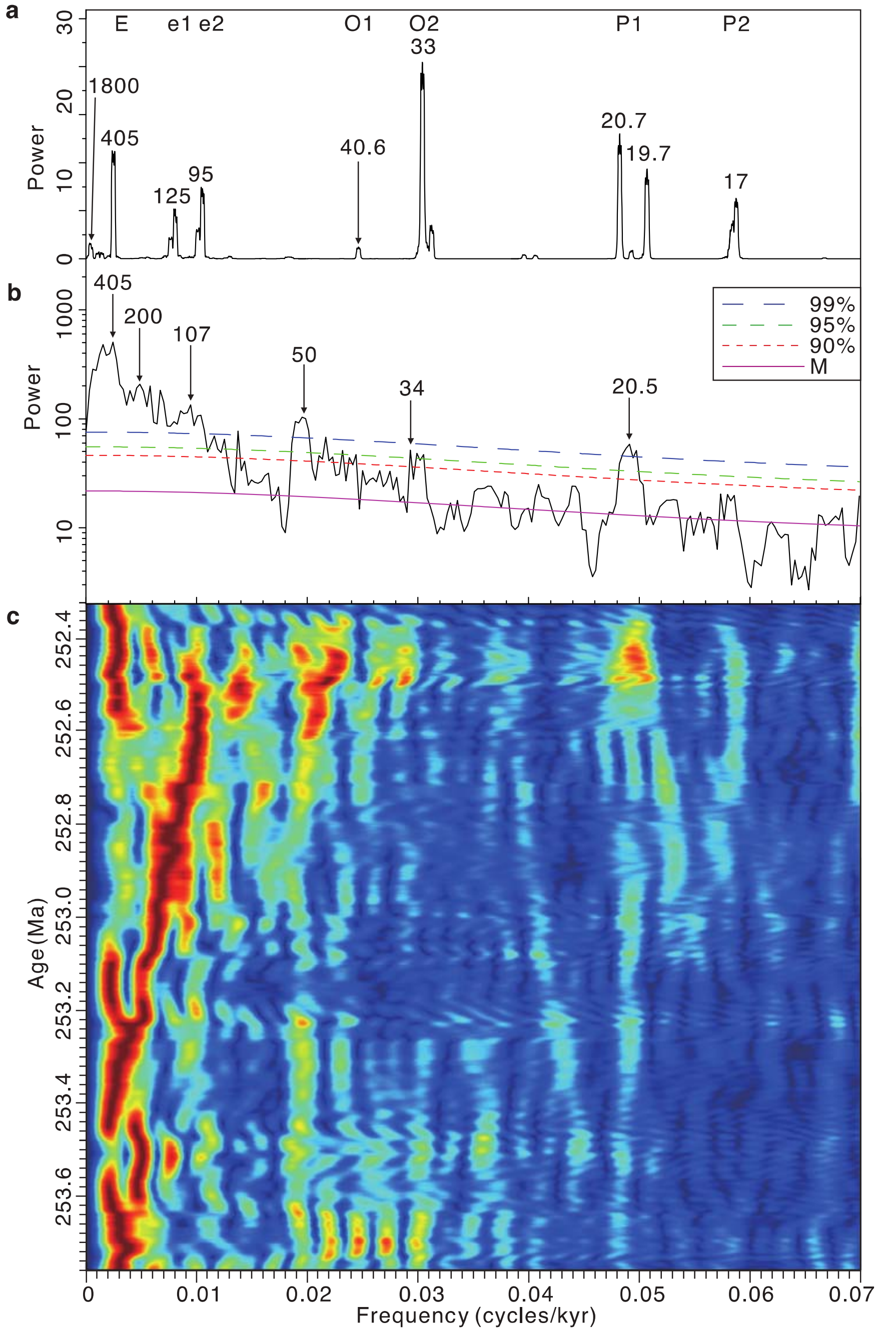

Supplementary Figure S9 | Meishan time series and La2004 spectra. (**a**) 2π MTM power spectrum for standardized, stacked La2004 eccentricity, obliquity and precession ("ETP") of 240-249 Ma. (**b**) 2π MTM power spectrum of the Meishan MS U-Pb age-calibrated time series. (**c**) Evolutionary spectrum for the U-Pb age-calibrated MS time series of the Meishan section, using a 500-kyr sliding window. Significant peaks are labeled in kyr. Legends as in Figure S8.

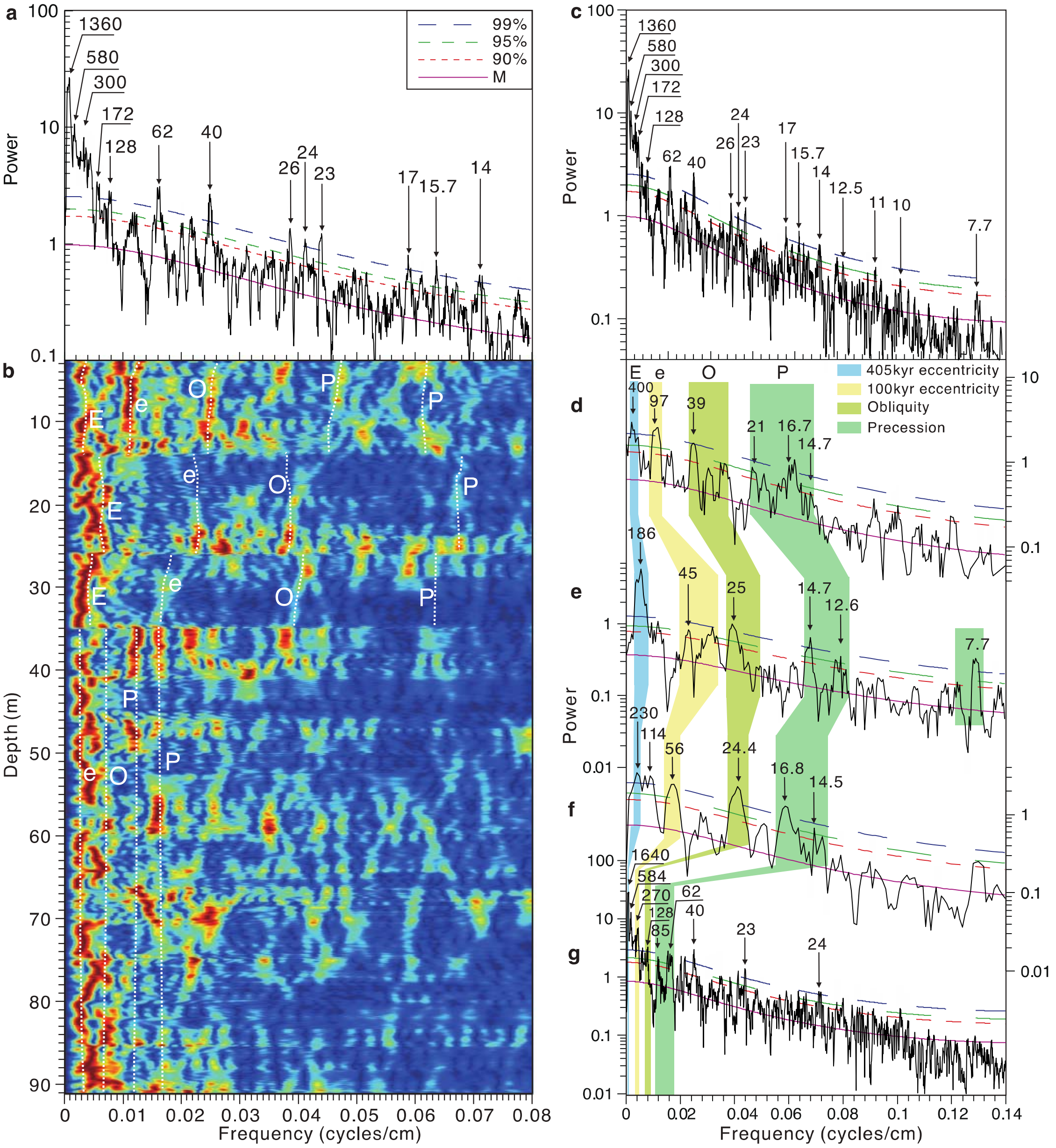

Supplementary Figure S10 | The Shangsi ARM stratigraphic series spectrum. (**a**) 3π MTM power spectrum, with significant peaks labeled in cm. The purple, red, green and blue (dashed) curves represent the median smoothed, linear fitted red noise spectrum, and 90%, 95% and 99% confidence levels. (**b**) Evolutionary FFT spectrum, with a 6 m sliding window. The dashed white line labeled with E, e, O and P represent the 405-kyr long eccentricity, 100-kyr short eccentricity, obliquity and precession cycles, respectively. (**c**) Spectrum as in (**a**), but over a wider frequency range. (**d-g**) 3π MTM power spectra of the ARM stratigraphic series subsets of 1-14 m, 14-26 m, 26-33 m and 33-93.6 m, respectively. Significant peaks are labeled in centimeters.

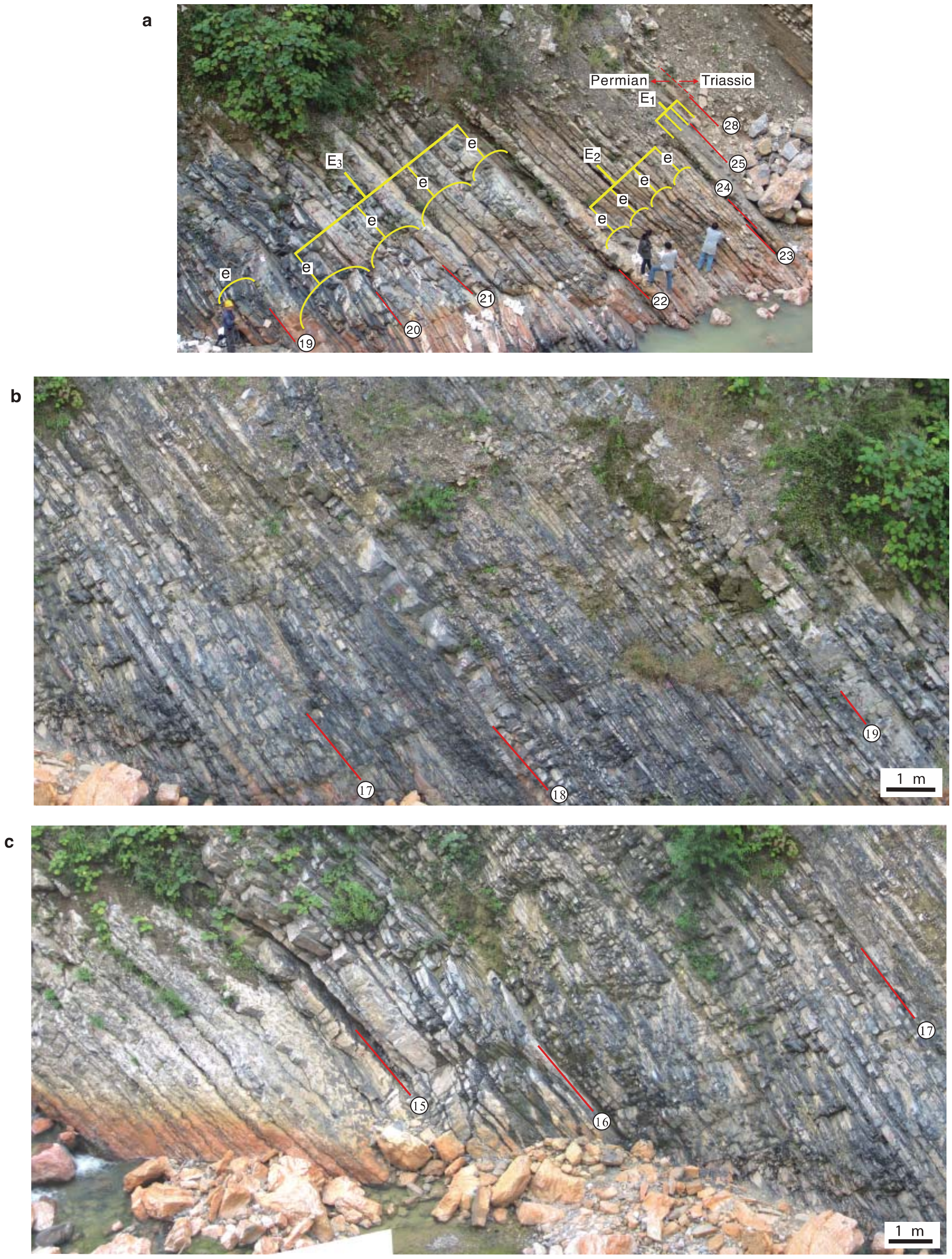

Supplementary Figure S11 | Outcropping section at Shangsi, China. (**a**) Photo of the upper part (Beds 19-28) of the Changhsingian Dalong Formation. Five thin precession-scale beds are bundled into 100-kyr eccentricity cycles (e), and four 100-kyr cycles are bundled into 405-kyr eccentricity cycles (E). The ARM cycle interpretation is provided in Fig. 2, Supplementary Figs. S4, S5 and S7. Eccentricity maxima are recorded by pronounced precession beds while the eccentricity minima correlate to thick limestone beds (see main text). (**b**) Photo of upper Bed 16 to lower Bed 19. (**c**) Photo of Beds 14 to 17 in the Dalong Formation. Beds 16 to 18 are composed of limestone and black shales or mudstone, and record lower sedimentation rates compared to Beds 19 to 22. Circled numbers indicate the bed numbers and red lines mark the bed boundaries.

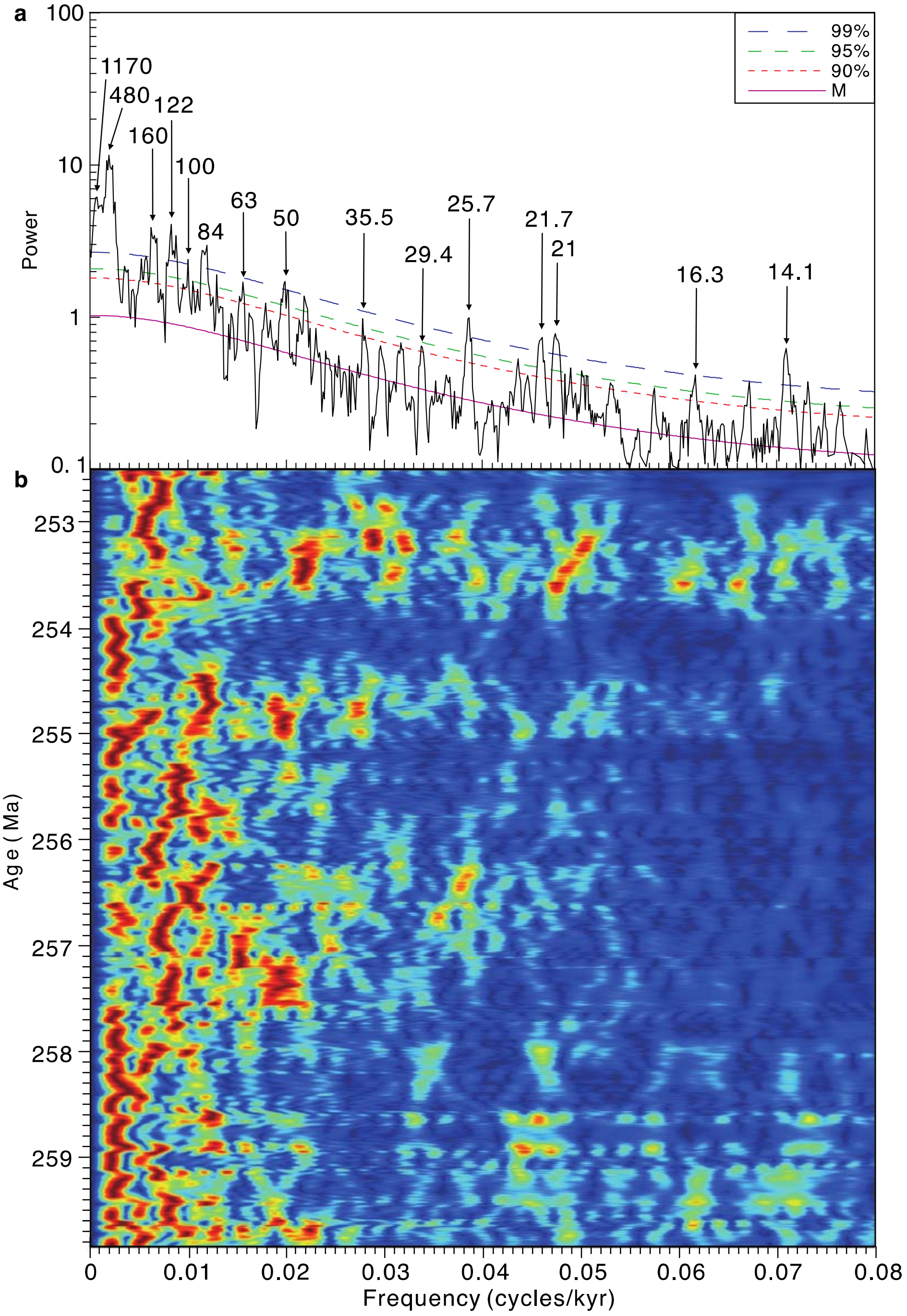

Supplementary Figure S12 | The U-Pb age-calibrated Shangsi ARM time series spectrum. (**a**) 3π MTM power spectrum. (**b**) Evolutionary FFT spectrum, using a 500-kyr sliding window. Significant peaks are labeled in kiloyears. Legends are as in Figure S8.

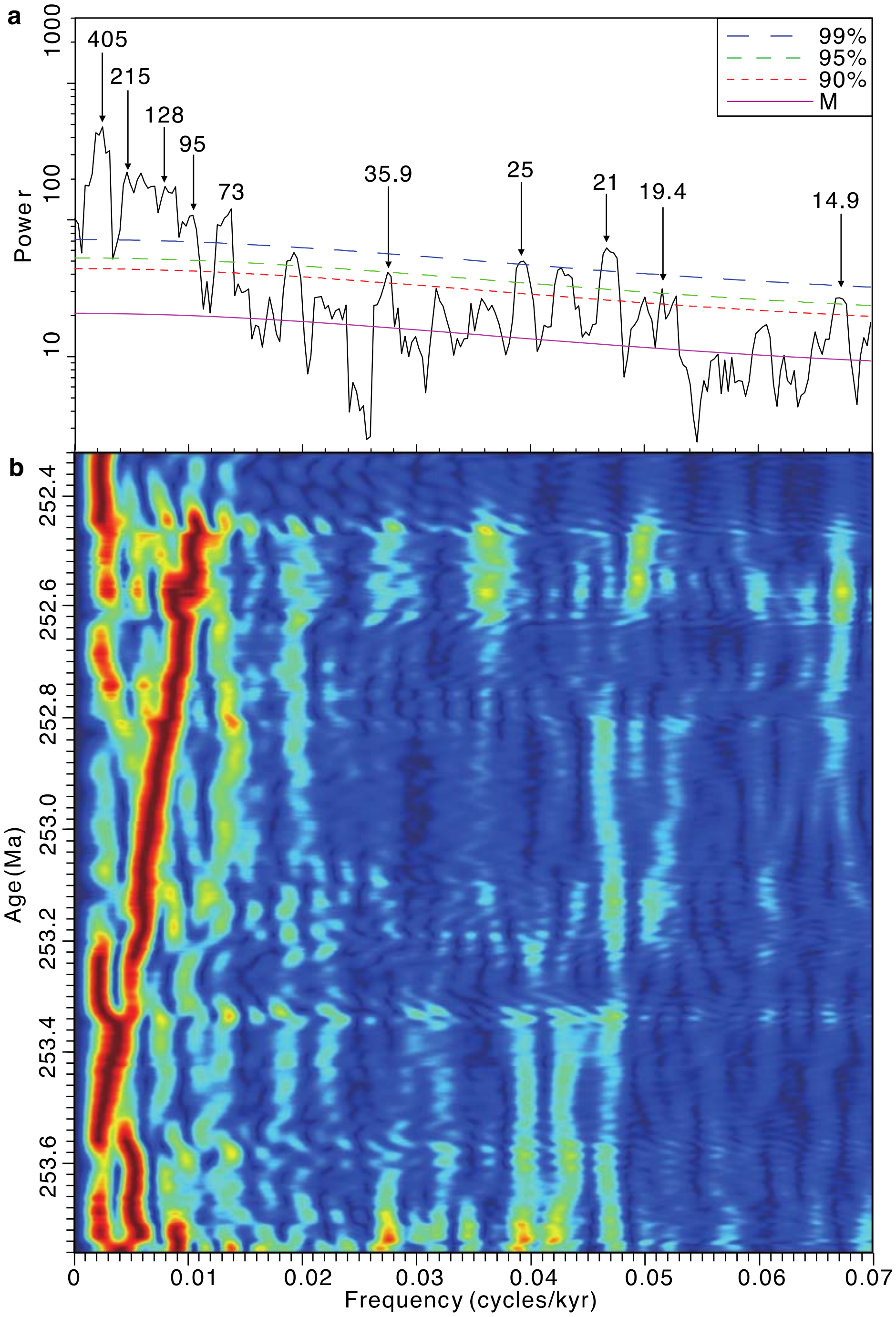

Supplementary Figure S13 | The 405-kyr tuned Meishan MS time series spectrum. (**a**) 2π MTM power spectrum. (**b**) evolutionary FFT spectrum, using a 500-kyr sliding window. Significant peaks are labeled in kiloyears.

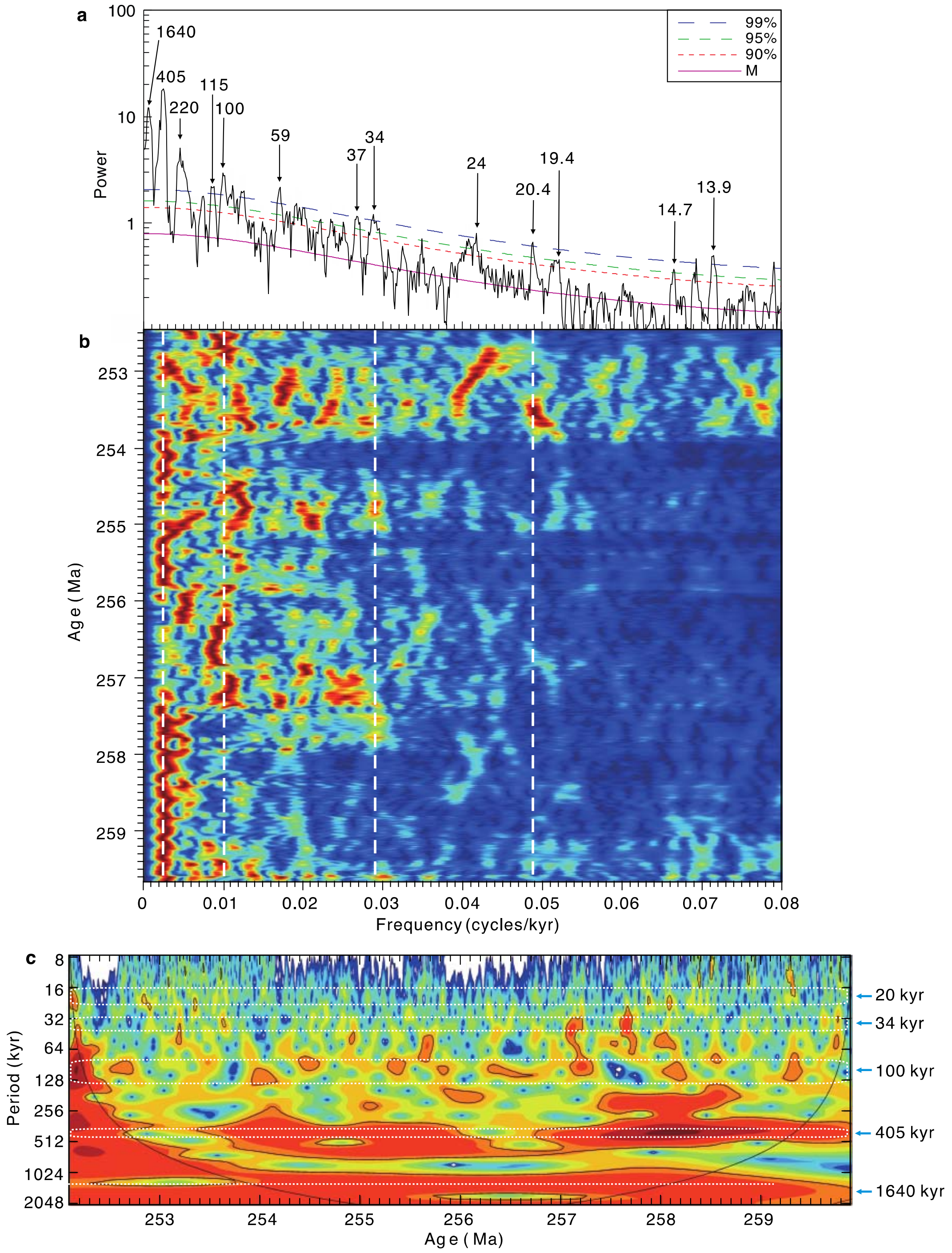

Supplementary Figure S14 | The 405-kyr-tuned Shangsi ARM time series spectrum. (**a**) 3π MTM power spectrum. (**b**) Evolutionary FFT spectrum, using a 500-kyr sliding window. Significant peaks are labeled in kiloyears. Legends are as in Figure S8. **(c)** Wavelet scalogram of the 405-kyr-tuned ARM time series. The shaded contours in wavelet scalogram are normalized linear variances, with blue representing low spectral power and red representing high spectral power. Regions below curves on both ends indicate the cone of influence where edge effects become significant.

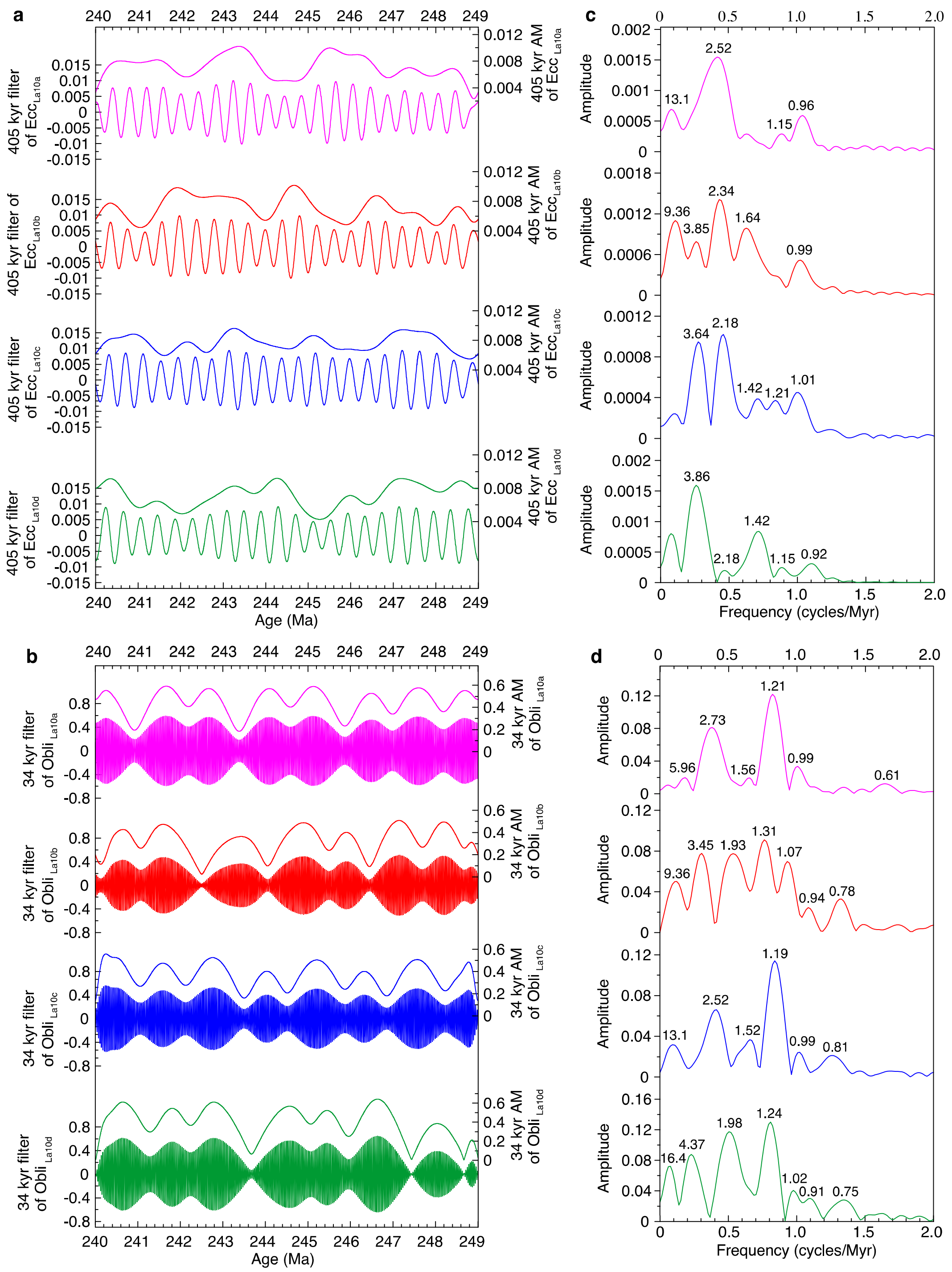

Supplementary Figure S15 | Long-period amplitude modulations (AM) of the 405 kyr eccentricity and obliquity of La2010 astronomical solutions (ref. 11). (a) 405 kyr eccentricity curves and AM, and **(b)** filtered 34 kyr bands and AM of the La10a (purple), La10b (red), La10c (blue) and La10d (green) in 240 – 249 Ma. The 405 kyr cycles and 34 kyr cycles were extracted with Taner passband filters (f_{low}, f_{center}, f_{high} in cycles/kyr) of (0.00146858, 0.00246858, 0.00346858) and (0.0266, 0.0291, 0.0316), respectively. (**c, d**) Corresponding 2π multitapered amplitude spectra of AM of 405 kyr cycles and 34 kyr obliquity cycles of La10a (purple), La10b (red), La10c (blue) and La10d (green). AM period peaks are labeled in Myr. The La2010 obliquity model was computed according to the procedure described in the appendix of Wu et al. (ref. 46).

Supplementary Tables

Supplementary Table S1 | Stratigraphic depth versus time in kiloyears (accumulation rates).

a. Meishan Section

Depth (m)	Age (ka)	Age (ka) anchored to 252.28 ± 0.13 Ma (ref. 12)	Sediment accumulation rates (cm/kyr)
-0.10	203		
0.21	304	252307	0.31
1.17	405	252408	0.95
8.87	810	252813	1.90
20.61	1215	253218	2.90
33.12	1620	253623	3.09
40.06	2025	254028	1.71
41.55	2126	254129	1.48

b. Shangsi section

Depth (m)	Age (ka)	Age (ka) anchored to 252.28 ± 0.08 Ma (ref. 12)	Sediment accumulation rates (cm/kyr)
-1.67	405	251792	
0.00	810	252197	0.41
0.98	1215	252602	0.24
3.94	1620	253007	0.73
10.44	2025	253412	1.60
13.64	2430	253817	0.79
16.70	2835	254222	0.76
17.87	3240	254627	0.29
19.16	3645	255032	0.32
20.48	4050	255437	0.33
22.25	4455	255842	0.44
23.23	4860	256247	0.24
24.36	5265	256652	0.28
25.96	5670	257057	0.39
27.54	6075	257462	0.39
29.53	6480	257867	0.49
33.84	6885	258272	1.06
46.94	7290	258677	3.23
64.54	7695	259082	4.34
77.62	8100	259487	3.23
93.56	8505	259892	3.94

Supplementary Table S2 | Ages and durations constrained of geological and biological events according to U-Pb ages and the 405-kyr-tuned ATS.

(a) Meishan section. The adjusted 405 kyr-tuned ages were obtained by shifting 134 kyr forward to match the astronomical chronology at Shangsi.

Stage	Formation	Conodont zones	Lower depth (m)	Lower age (ka)			Duration (kyr)	
				U-Pb age (ref.12)	405-kyr -tuned age	Adjusted 405-kyr -tuned age	U-Pb age (ref. 12)	405-kyr tuned age
		I. isarcica	-0.06	252130	252215	252081		
		I. staeschei	-0.03	252150	252224	252090	20	9
Induan		*H. parvus*	0.00	252172	252234	252100	22	10
	Yingkeng		0.16	252287	252283	252149	-	-
Chang-hsingian			41.16	253911	254104	253970	1739	1870
	Changxing		41.95	253979	254158	254024	1692	1875
		C. meishanensis	0.16	252287	252283	252149	115	49
		C. yini	3.10	252412	252511	252377	125	228
		C. changxingensis	25.00	253106	253361	253227	694	850
		C. subcarinata	34.05	253396	253679	253545	290	318
		C. wangi	41.16	253911	254104	253970	515	425
Wuchia-pingian		*C. orientalis*	-					
	Longtan		-					

(b) Shangsi Section

Stage	Formation	Conodont zones	Lower depth (m)	Lower age (ka)		Duration (kyr)	
				U-Pb age (ref.12)	405-kyr-tuned age	U-Pb age (ref. 12)	405-kyr-tuned age
Induan		*H. eurypyge*	-0.4	252178	252100		
	Feixianguan		0	252246	252197		
Changhsingian			15.9	254113	254115	1935	2015
	Dalong		42.02	258296	258523	6050	6326
		C. meishanensis	0.1	252264	252239	86	139
		C. yini	2.7	253060	252837	796	598
		C. changxingensis	9.05	253410	253325	350	488
		C. subcarinata	10.6	253488	253431	78	106
		C. wangi	15.9	254113	254115	625	684
Wuchiapingian			-				
		C. orientalis	22.2	256016	255828	1903	1713
		C. transcaucasica	-	-	-		
	Wujiaping		-				
